# Antimicrobial Activities and Mechanisms of Magnesium Oxide Nanoparticles (nMgO) against Pathogenic Bacteria, Yeasts, and Biofilms

**DOI:** 10.1038/s41598-018-34567-5

**Published:** 2018-11-02

**Authors:** Nhu-Y Thi Nguyen, Nathaniel Grelling, Cheyann Lee Wetteland, Romeo Rosario, Huinan Liu

**Affiliations:** 1 Microbiology Graduate Program, University of California, Riverside, CA 92521 USA; 2 Department of Bioengineering, University of California, Riverside, CA 92521 USA; 3 Materials Science and Engineering Program, University of California, Riverside, CA 92521 USA

## Abstract

Magnesium oxide nanoparticle (nMgO) is a light metal based antimicrobial nanoparticle that can be metabolized and fully resorbed in the body. To take advantage of the antimicrobial properties of nMgO for medical use, it is necessary to determine the minimal inhibitory, bactericidal and fungicidal concentrations (MIC, MBC and MFC) of nMgO against prevalent infectious bacteria and yeasts. The objective of this study was to use consistent methods and conditions to reveal and directly compare the efficacy of nMgO against nine prevalent pathogenic microorganisms, including two gram-negative bacteria, three gram-positive bacteria with drug-resistant strains, and four yeasts with drug-resistant strains. The MIC of nMgO varied from 0.5 mg/mL to 1.2 mg/mL and the minimal lethal concentration (MLC) of nMgO at 90% killing varied from 0.7 mg/mL to 1.4 mg/mL against different pathogenic bacteria and yeasts. The most potent concentrations (MPC) of nMgO were 1.4 and/or 1.6 mg/mL, depending on the type of bacteria and yeasts tested. As the concentration of nMgO increased, the adhesion of bacteria and yeasts decreased. Moreover, *S. epidermidis* biofilm was disrupted at 1.6 mg/mL of nMgO. *E. coli* and some yeasts showed membrane damage after cultured with ≥0.5 mg/mL nMgO. Overall, nMgO killed both planktonic bacteria and disrupted nascent biofilms, suggesting new antimicrobial mechanisms of nMgO. Production of reactive oxygen species (ROS), Ca^2+^ ion concentrations, and quorum sensing likely contribute to the action mechanisms of nMgO against planktonic bacteria, but transient alkaline pH of 7 to 10 or increased Mg^2+^ ion concentrations from 1 to 50 mM showed no inhibitory or killing effects on bacteria such as *S. epidermidis*. Further studies are needed to determine if specific concentrations of nMgO at MIC, MLC or MPC level can be integrated into medical devices to evoke desired antimicrobial responses without harming host cells.

## Introduction

Infection is a major clinical complication associated with implanted medical devices, which costs 5–10 billion dollars per year to treat, prolongs the hospital stay, and causes clinical complications for patients^1^. Sixty to seventy percent of all nosocomial (hospital-acquired) infections involve biofilms, and biofilms are 1000-fold more resistant to antibiotics than planktonic bacteria^2,3^. Systemically administered antibiotics and antifungals are used to treat infections, but they often cannot penetrate biofilms and more bacteria and fungi become drug resistant^4,5^. Once infected, the medical devices and implants often require a secondary surgery or procedure for removal, which increases healthcare cost and discomfort for patients^6–8^. Considering that insurance programs such as Medicare ceased payments for hospital-acquired infections in 2008^9^, the costs for hospitals and patients further escalated.

New biomaterials are critically needed to reduce or eliminate microbial adhesion and infections, thus minimizing the use of antibiotics and emergence of antibiotic-resistant strains and mitigating infections of medical devices. Many nanoparticles such as silver nanoparticles, zinc oxide (ZnO) nanoparticles, and titanium oxide (TiO_2_) nanoparticles have shown antimicrobial properties against a broad spectrum of microorganisms^10–13^. However, these nanoparticles cause significant concerns regarding their toxicity due to the risks associated with heavy metal elements and their accumulation in the body. In contrast, magnesium oxide nanoparticles (abbreviated as nMgO) is an attractive alternative to heavy metal based nanoparticles such as silver and ZnO, because nMgO can be degraded and metabolized efficiently in the body, and the released degradation products of Mg^2+^ and OH^−^ ions can be effectively eliminated from the body as long as renal function is normal, thus removing the concerns of excessive metal accumulation in the body.

Previously, MgO nanoparticles (nMgO) have been reported to inhibit gram-positive, gram-negative, and endospore-forming bacteria^14–18^. Tang *et al*. have reviewed MgO nanoparticles as antibacterial agent^19^. However, there are no standards established for testing the antimicrobial properties of nanoparticles. Thus, different experimental techniques were used, MgO with varying sizes and concentrations were studied, and different initial seeding densities of bacteria were used in the previous studies on antimicrobial properties of MgO. These experimental differences do affect the results significantly, which made the previous results not directly comparable with one another and not directly applicable for designing antimicrobial medical devices. Even though the previous studies did prove that MgO had antibacterial properties under different conditions, the antimicrobial potency of nMgO is still unknown and incomparable against a wide spectrum of pathogenic microorganisms. It is necessary to establish consistent conditions in order to directly compare the effects of the same nMgO dosages on a wide range of clinically relevant microorganisms. In order to take full advantage of nMgO toward potential clinical translation to broad medical applications, a consistent method was established in this study and used to determine the efficacy of nMgO against different microorganisms. Under the consistent conditions, we investigated and directly compared the effects of nMgO on nine different types of pathogenic microorganisms in planktonic forms or biofilms, including gram-negative bacteria, gram-positive bacteria, yeasts, and their resistant strains. According to our literature search, this is the first study to use the same well-defined and consistent method to quantify and directly compare antimicrobial activities of nMgO against five major infectious bacteria with drug resistant strains, four yeasts with drug resistant strains, and nascent biofilms. Moreover, this is the first time to study the nMgO activity against *Candida glabrata* (*C. glabrata)*, an organism that has been gaining resistance to multiple widely-used antifungals^20^.

The first objective of this study was to use the consistent method to examine, quantify and compare the concentration effects of nMgO against the following microorganisms. Specifically, two gram-negative bacteria include *Escherichia coli* (*E. coli*) and *Pseudomonas aeruginosa* (*P. aeruginosa*); three gram-positive bacteria include *Staphylococcus epidermidis* (*S. epidermidis*), *Staphylococcus aureus* (*S. aureus)*, and methicillin-resistant *Staphylococcus aureus* (MRSA); and four infectious yeasts include drug-sensitive *Candida albicans* (*C. albicans*), Fluconazole resistant *Candida albicans* (*C. albicans FR)*, drug-sensitive *Candida glabrata* (*C. glabrata*), and echinocandin resistant *Candida glabrata* (*C. glabrata ER*). The minimal inhibitory concentration (MIC) of any antibiotic or antifungal drugs is the lowest concentration required to inhibit visible growth of a microorganism in culture. The minimal lethal concentration (MLC) is the lowest concentration of any antibiotic or antifungal drugs required to kill a microorganism in culture, that is, no growth of the microorganism even when it is sub-cultured subsequently in drug-free media^21^. The minimal bactericidal or fungicidal concentration (MBC or MFC) is the MLC for bacteria or yeasts respectively, i.e., the lowest concentration required to kill a respective bacterium or fungus in culture. MBC and MFC are generally considered as the dosage at which 99.9% of bacteria or yeasts are killed^22^, however, some also used 90% of death as a cut off ^23,24^. The same concept of MIC, MLC, MBC, and MFC was adapted for the respective concentrations of nMgO in this article. However, it is important to mention that MgO is not considered as an antibiotic or antifungal drug. The MLC_90_, MLC_99_, MLC_99.9_, and MLC_99.99_ were used in this article to refer to the MBC or MFC of nMgO that killed 90%, 99%, 99.9% or 99.99% of bacteria or yeasts during 24 hours of culture *in vitro*.

The nine microorganisms above were chosen because they are prevalent pathogenic species isolated from infected medical devices and represent a large variety of bacteria and yeasts, including the drug-resistant strains^6,25,26^. Drug resistant strains are included in this study because they are major threats in the health care, and it is clinically important to determine and compare the efficacy of nMgO against the antibiotic/antifungal resistant microorganisms. The concentrations of nMgO between 0.2 mg/mL and 2.0 mg/mL were chosen because this range covered different concentrations that have shown antimicrobial activities in the literature^15,17,18,27^. The concentrations of nMgO below 0.2 mg/mL were not included in this study because the lower concentrations have no detectable antimicrobial effects on the microbes of interest. Moreover, Wetteland *et* al. studied the interactions of 0.2–2.0 mg/mL nMgO with bone marrow derived mesenchymal stem cells (BMSCs)^18^; and the same concentration range of nMgO was used against microbes in this study in order to compare with the previous results. Since planktonic bacteria have to adhere to a material or medical device to initiate an infection, the second objective was to evaluate the adhesion and morphologies of different microorganisms after they were cultured with 0–2.0 mg/mL of nMgO. Considering that the established biofilms are much harder to treat, the third objective was to determine if the nMgO can disrupt a model biofilm of *S. epidermidis* at the concentration of nMgO that showed the highest potency against *S. epidermidis*. The possible interaction mechanisms of nMgO with these pathogenic bacteria, yeasts, and biofilms were discussed.

## Materials and Methods

### Prepare and Characterize MgO nanoparticles (nMgO)

MgO nanoparticles (nMgO; US Research Nanomaterials Inc.) had a purity of 99+%, with an average diameter of 20 nm and polyhedral morphology. MgO has a true density of 3.58 g/cm^3^. For this study, MgO nanoparticles were sterilized through heating in an oven at 200 °C for one hour before each *in vitro* experiment, because other methods of sterilization or disinfection are not suitable for nMgO. Specifically, nMgO is hygroscopic; and, thus, autoclaving is not an ideal method for sterilization, because the water in the steam causes phase change, which affects the accuracy of experimental results against microorganisms. Furthermore, nMgO absorbs ultraviolet (UV) light and interacts with oxygen molecules^18,28^; and, thus, UV should not be used to disinfect the nMgO nanoparticles.

MgO nanoparticles were characterized using scanning electron microscopy (SEM, Nova NanoSEM 450) and transmission electron microscopy (TEM, Titan Themis 300). The particle size and distribution were analyzed using the quantitative tools in the ImageJ software. Elemental composition and phase of these nanoparticles were confirmed using energy dispersive x-ray spectroscopy (EDS, EDAX Leap detector attached to Nova NanoSEM), and x-ray diffraction (XRD, Model D8/Advanced, Bruker), respectively.

The zeta potential of nMgO in water was measured following a method adapted from Tian *et al*.^29^. Briefly, a zeta potential and submicron particle size analyzer (Delsa™ Nano C, Beckman Coulter) was used to determine the zeta potential of nMgO. Water was used as the diluent, which had a refractive index of 1.33, viscosity of 0.89 cP, and dielectric constant of 78.3. The test was carried out at the room temperature of 25 °C with an applied potential of 5 V/cm, and was repeated for three times. The electrical mobility of nMgO was measured based on the frequency shift of the scattered light using the Laser Doppler method, and the zeta potential of nMgO was calculated according to Huckel equation.

### Bacterial and yeast cultures

The stocks of *P. aeruginosa*, *S. aureus*, MRSA, *C. albicans, C. albicans FR, C. glabrata* and *C. glabrata ER* were generously provided by Dr. Jill P. Adler-Moore from California State Polytechnic University, Pomona.

#### Culture methods for gram-positive and gram-negative bacteria

Frozen stocks of *E. coli* (ATCC 25922) and *Pseudomonas aeruginosa* (*P. aeruginosa*) (ATCC 29260) were retrieved from the −80 °C freezer, and cultured in Luria Bertani Broth (LBB; Sigma Life Science, Sigma-Aldrich) and Tryptic Soy Broth (TSB; Fluka Analytical, Sigma-Aldrich), respectively. Five milliliters of the frozen stock were cultured in 30 mL of the respective broth for 16 hours in a shaker incubator (Benchmark Incu-shaker 10L, VWR) at 37 °C and 250 rpm. The concentrations of *E. coli* and *P. aeruginosa* were determined by counting bacteria with a hemocytometer (Brightline, Hausser Scientific) and they were diluted to 7.8 × 10^6^ cells/mL in LBB or TSB.

Similarly, frozen stocks of *S. epidermidis* (ATCC 35984), *S. aureus* (ATCC 25923), and MRSA (ATCC 33591) were retrieved and cultured in TSB overnight in a shaker incubator at 37 °C and 250 rpm. The next day, 100 µL of the culture was added into 5 mL of fresh TSB, and this culture was incubated for additional 4–6 hours at 37 °C. After that, the concentrations of bacteria were determined using a hemocytometer and they were diluted to 7.8 × 10^6^ cells/mL using TSB.

#### Culture methods for drug-sensitive and drug-resistant yeasts

Frozen stocks of *C. albicans* (CP 620) and *C. albicans FR* (CP 714) were placed into Sabouraud Dextrose broth (Sab; Difco, VWR) in a 50 mL centrifuge tube. Five milliliters of the yeasts from the stock were cultured in 30 mL of Sab for 22 hours at 37 °C. After 22 hours, 2 mL of yeasts were transferred to 30 mL fresh Sab and cultured for 20 hours at room temperature. After that, 2 mL of yeast was transferred again to fresh Sab and cultured for 16 hours at room temperature. Three days of culture in different conditions ensured that yeast cells were in the single-cell form and did not have pseudohyphae at the start of each *in vitro* experiment. Pseudohyphae are long chains of budding yeast daughter cells that did not totally separate from the mother cells, mimicking a hyphae^30^.

Frozen stocks of *C. glabrata* (ATCC 90030) and *C. glabrata ER* (17351) were also cultured in Sab broth. They were incubated for 24 hours at 37 °C without subsequent subculture. It was not necessary to subculture these yeasts because *C. glabrata* does not form pseudohyphae.

#### Prescribed seeding density of 7.8 × 10^6^ cells/mL for the bacteria and yeasts of interest

Each bacterial strain was cultured to the exponential phase as described above. In order to determine the concentration of respective microorganism in the broth before each *in vitro* experiment, the bacteria and yeasts were counted using a hemocytometer^31,32^. For this purpose, each culture was diluted to 1:100 using a Tris(hydroxymethyl)aminomethane buffer (Tris buffer; Acros, Sigma-Aldrich). Ten microliters of the diluted bacterial suspension were loaded onto each side of the hemocytometer. The suspension was left to settle for 2 to 3 minutes for motile bacteria such as *E. coli* and *P. aeruginosa*. The bacteria were counted in the regions of the 25 squares in the center of hemocytometer using a compound microscope (VEEVanGuard, Biosciences) at a magnification of 400x. Only four corner squares and the center square were counted. After both sides of the hemocytometer were counted, the following equation was used to calculate the bacterial density:

$$\begin{array}{rcl}{\rm{Bacterial}}\,{\rm{density}} & = & ({\rm{bacterial}}\,{\rm{counts}}\,{\rm{on}}\,{\rm{both}}\,{\rm{sides}}\,{\rm{of}}\,{\rm{the}}\,{\rm{hemocytometer}}\\  &  & /2)(5)({\rm{dilution}}\,{\rm{factor}})({{\rm{10}}}^{{\rm{4}}}\,{\rm{mL}})\end{array}$$

The next step was to dilute the bacteria to the desired concentration of 7.8 × 10^6^ cells/mL using either LBB or TSB according to C_1_V_1_ = C_2_V_2_ equation. This was the working stock for *in vitro* experiments.

Counting yeasts was similar as counting bacteria, except one additional step. Yeasts are eukaryotic cells, and are much larger than bacteria. We used methylene blue stain (Fisher Chemicals, Fisher Scientific) to make sure we only counted live yeast cells. After the yeasts were cultured to the exponential phase, they were diluted to 1:5 using a Tris buffer and 20 µL of methylene blue stain. The counting and calculation were the same as described above for the bacteria. The yeasts were diluted with Sab to the desired concentration of 7.8 × 10^6^ cells/mL for all *in vitro* experiments. The seeding density for yeasts was the same as for bacteria in order to directly compare the results between bacteria and yeasts.

#### Verify the actual seeding density used for each microorganism of interest

After the bacteria and yeasts were diluted to the desired concentrations of 7.8 × 10^6^ cells/mL, an extra step was taken to confirm the actual seeding density for each microorganism. Each of the working stocks of bacteria or yeasts was plated onto its respective agar plate, either Luria Bertani agar (LB agar; Sigma Aldrich), Tryptic soy agar (TSA; Fluka Analytical, Sigma Aldrich) or Sabouraud agar (Sab agar; Gibco, VWR). All of the working stocks were diluted in a series of tenfold dilutions to reach 10^–4^, and 100 µL was plated onto the agar. These plates were incubated at 37 °C for 24 hours, and the colony forming units (CFU) on the plates were counted to calculate the actual seeding density. The prescribed seeding density of 7.8 × 10^6^ cells/mL was based on the counting with the hemocytometer, but the actual seeding density fluctuated between 6 × 10^6^ and 8 × 10^6^ CFU/mL as determined by the actual counting of CFUs on the respective agar plates.

### Determine the concentration effects of nMgO against the bacteria and yeasts of interest

#### Culture bacteria and yeasts with 0.2 mg/mL to 2.0 mg/mL of nMgO

The nMgO powder was dehydrated and sterilized in an oven at 200 °C for at least one hour before each *in vitro* experiment. In order to obtain the prescribed concentrations of 0.2 mg/mL to 2.0 mg/mL of nMgO, 0.6–6.0 mg of sterilized nMgO particles were weighed in the sterile 2 mL microcentrifuge tubes using an analytical balance (MS105D, Mettler Toledo) prior to the *in vitro* cultures with bacteria or yeasts. Three milliliters of the respective working stocks of diluted bacteria or yeasts with a concentration of 6 × 10^6^ to 8 × 10^6^ cells/mL were transferred into the respective wells in the non-treated 12-well culture plates. After the 3 mL of the respective bacterial or yeast suspension was transferred into the culture wells, 1 mL was placed into the respective microcentrifuge tubes to collect the previously weighed nMgO particles and transfer them into the respective wells of the 12-well plates. The culture plates were incubated for 24 hours in a shaker incubator at 37 °C and 120 rpm. The shaking speed was reduced from the initial 220 rpm for bacterial or yeast culture to prevent the microorganisms in one well spilling into neighboring wells. Experimental and control samples were all run in triplicates.

#### Quantify the concentration and viability of bacteria and yeasts after cultured with 0.2 mg/mL to 2.0 mg/mL of nMgO

After 24 hours of culture with nMgO, the concentration and viability of the respective bacteria and yeasts were determined through plating and counting CFUs on their respective agar plates. Specifically, the broths containing the respective microorganism and the nMgO were collected into 15 mL conical tubes and vortexed. After that, 500 µL was aliquoted into a 2 mL microcentrifuge tube and serially diluted in a Tris buffer. To quantify the CFUs, 100 µL of the diluted or non-diluted suspension was spread onto the respective agar plates for that type of bacterium or yeast. The colonies on the agar plates were counted after they were incubated for 16–24 hours in a 37 °C incubator.

#### Evaluate the effects of nMgO on adhesion and morphology of the microorganisms of interest

In order to evaluate the possible changes in cell adhesion and morphology, the respective bacteria and yeasts were cultured on a glass square with nMgO in the range of 0–2.0 mg/mL, using the same method as described above. Specifically, the borosilicate microscope cover glass (Fisherbrand, Fisher Scientific 12-542-B) were cut into 1 cm × 1 cm squares, cleaned with acetone and ethanol sequentially under sonication for at least 30 min and sterilized in autoclave, and then placed into each culture well before adding the respective microorganisms and nMgO. The glass squares were used as a standard substrate for observing adhesion and morphology of each microorganism under scanning electron microscopy (SEM), because glass is a reproducible and widely-accepted reference for *in vitro* cultures involving mammalian cells and microorganisms. Each reference glass used here was thoroughly cleaned and sterilized to ensure that it does not induce random variables affecting the interactions of nMgO with the microorganisms.

After 24 hours of culture, the broths including the nMgO were collected, and the glass squares were rinsed three times with the Tris buffer. After the third wash, the respective microorganisms on the glass were fixed with 10% glutaraldehyde (Sigma Life Sciences, Sigma Aldrich) for one hour. After fixation, the samples were rinsed with a Tris buffer again three more times to wash away residual glutaraldehyde. These samples were dried in air at room temperature for at least 24 hours. The dried samples were sputter coated (108 Auto Sputter, Cressington) with Pt/Pd before SEM imaging. The SEM images were taken with backscattered electron detector, at an accelerating voltage of 10 kV and working distance of 5 mm. Bacteria were imaged at an original magnification of 5000x and the yeasts were imaged at the original magnifications of 500x and 5000x.

#### Analyze the broth pH and ion concentrations after culturing microorganisms with nMgO

After 24 hours of culture with nMgO, the collected broths were analyzed for pH using a calibrated pH meter (Symphony, VWR); and the concentrations of Mg^2+^ and Ca^2+^ ions in the collected broth were measured using inductively coupled plasma - optical emission spectrometry (ICP-OES; Optima 8000, Perkin Elmer). The concentrations of calcium (Ca^2+^) ions were measured in addition to the concentrations of Mg^2+^ ions because both Mg^2+^ and Ca^2+^ ions are relevant for cellular processes and nMgO was shown to affect the concentrations of both Mg^2+^ and Ca^2+^ ions in the culture media^18^. The broths were first diluted with deionized water prior to the ICP-OES measurements of the ion concentrations. TSB was diluted to 1:20; and LBB and Sab broth were diluted to 1:50. Each sample was centrifuged to ensure that solid or debris from the culture was precluded from the ICP-OES measurements. The dilutions were performed one day before they were analyzed in the ICP-OES instrument.

### Investigate the effects of nMgO against biofilms

#### Establishing a biofilm and exposing to 1.6 mg/mL of nMgO

*S. epidermidis* was selected as a model bacterium for investigating the effects of nMgO against biofilms because this gram-positive bacterium is one of the leading infectious agent for biofilm formation on medical devices and implants^26,33^. Bacteria culture and glass substrates were prepared using the same methods as described above. The protocol for establishing *S. epidermidis* biofilm was adapted from literature^34^. Briefly, the bacteria were cultured overnight, diluted at a factor of 1:100 with TSB; and 3 mL of diluted suspension was aliquoted onto a clean sterile glass substrate placed in each well of a 12-well non-treated culture plate. The culture plate was incubated at 37 °C for 48 hours to form biofilms on the glass substrates in each well. Afterwards, the bacteria on glass substrates were taken out of some wells, and stained with crystal violet (CV; Fisher Scientific) and imaged under SEM to confirm that a biofilm had formed. After the *S. epidermidis* biofilm was established, 2 mL of broth was extracted and 2 mL of fresh TSB with or without nMgO was added into each well. For the experimental group with nMgO, TSB with 1.6 mg/mL nMgO was added. For the control group, only 2 mL of TSB was added without nMgO. Afterwards, the culture plate was incubated at 37 °C for another 24 hours.

#### Evaluating the biofilm disruption using crystal violet staining and SEM imaging

After the *S. epidermidis* biofilm was incubated with or without nMgO for 24 hours, each well was washed 3 times with Tris buffer to remove free nMgO and free bacteria, and then stained with 0.1% CV in Tris buffer. After staining for 30 minutes, the culture wells were washed three times with deionized water to remove the unbound CV. After washing, all the samples on glass substrates were transferred to a new plate; the CV bound to bacteria was extracted and collected by submerging each sample in the 0.5 mL of 95% ethanol for 30 minutes, since only CV dissolves in ethanol. The extracted CV from each well was quantified by reading the absorbance at 570 nm using a spectrophotometer (Tecan, Infinite M2000 pro). To visualize the biofilm under SEM, the bacteria and biofilm on glass substrates were washed and fixed with 10% glutaraldehyde following the same procedures of sample preparation for SEM imaging, as described above.

### Investigate the effects of increased pH and Mg^2+^ ion concentrations on bacteria

After the *in vitro* cultures of nMgO with the bacteria or yeasts for 24 hours, all the cultures showed increasing broth pH and Mg^2+^ ion concentrations due to dissociation of nMgO. The effects of elevated pH or Mg^2+^ ion concentrations on the viability of bacteria was studied using *S. epidermidis* as a model microorganism. To determine the effects of increasing pH on bacterial viability, *S. epidermidis* were cultured in TSB with the initial pH intentionally adjusted to 7–10 using sodium hydroxide (NaOH, STREM Chemicals). At the end of 24 hours of culture, the viability of bacteria was quantified by plating and counting CFUs on agar plates; and the post-culture broth pH and ion concentrations were analyzed using the same methods as described above.

To determine the effects of increasing Mg^2+^ ions on bacterial viability, *S. epidermidis* was cultured in TSB doped with supplemental Mg^2+^ ions. For this purpose, the stock of 150 mM of Mg^2+^ in broth were prepared by dissolving 1.525 g of magnesium chloride hexahydrate (MgCl_2_∙6H_2_O, Sigma Aldrich) in 50 mL of TSB. The 150 mM stock was then diluted with the broth to obtain the TSB with supplemental Mg^2+^ dosage that ranged from 1 to 50 mM. The supplemental Mg^2+^ dosage reported here did not include the baseline Mg^2+^ ions already present in the TSB. That is, 0 mM group means that no supplemental Mg^2+^ ion was added into the broth. At the end of 24 hours of culture, the viability of bacteria was quantified by plating and counting CFUs on agar plates; and the post-culture broth pH and ion concentrations were analyzed using the same methods as described above.

## Results

### Characterization of nMgO

**Figure 1:**
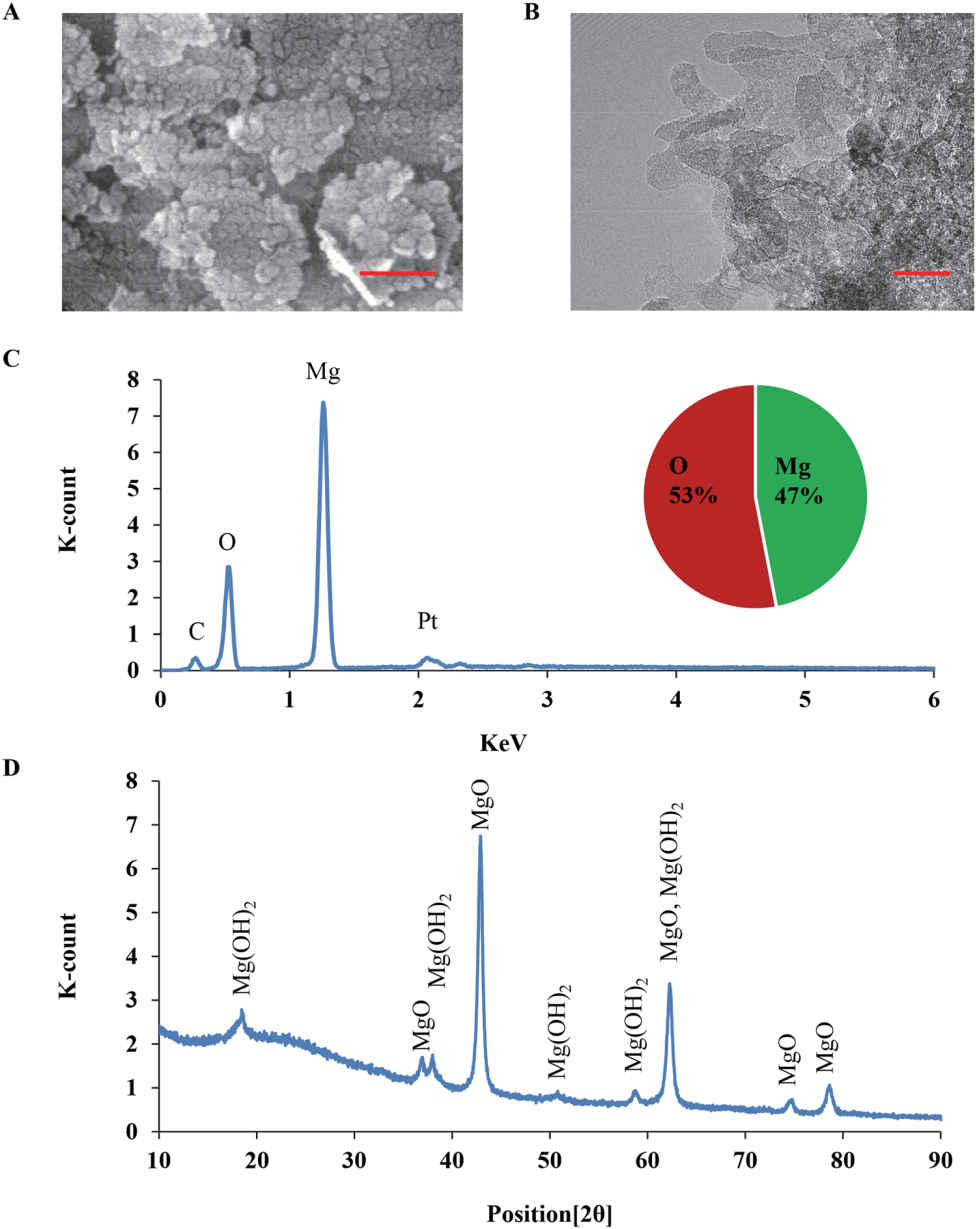
Characterization of microstructure, elemental composition, and phase of nMgO. (**A**) SEM image of nMgO at an original magnification of 250,000x. Scale bar: 200 nm. (**B**) TEM image of nMgO, Scale bar: 20 nm. (**C**) EDS analysis of nMgO. (**D**) XRD pattern of nMgO.

SEM and TEM images of nMgO confirmed its nanometer size and polyhedral morphology (Fig. 1A,B), which is in agreement with the information from the vendor. EDS analysis confirmed the elemental composition of the MgO nanoparticles containing magnesium (Mg) and oxygen (O), close to 1:1 atomic ratio (Fig. 1C). The expected crystalline phase of nMgO was confirmed with the standard peaks in the XRD pattern (Fig. 1D). The peaks for Mg(OH)_2_ were also present in the XRD spectrum, indicating the presence of Mg(OH)_2_ phase. The presence of Mg(OH)_2_ peaks in the XRD spectrum is expected, because MgO nanoparticles are hygroscopic and they can readily react with water in the atmosphere to form Mg(OH)_2_. The size distribution of nMgO particles was normal and narrow, with an average of 23 ± 5 nm, in agreement with the literature^18^. As listed in Table 1, the zeta potential and electrical mobility of nMgO in water were 32.31 ± 4.1 mV and 1.68 ± 0.22 (×10^−4^ cm^2^ V^−1^ s^−1^), similar to what were reported in literature^35^.

**Table 1:** Zeta potential and electrical mobility of nMgO in water.

Sample	Zeta Potential (mV)	Electrical mobility (× 10^−4^ cm^2^ V^−1^ s^−1^)
nMgO	32.31 ± 4.1	1.68 ± 0.22

### The concentration effects of nMgO on viability of major pathogenic bacteria and yeasts

**Figure 2:**
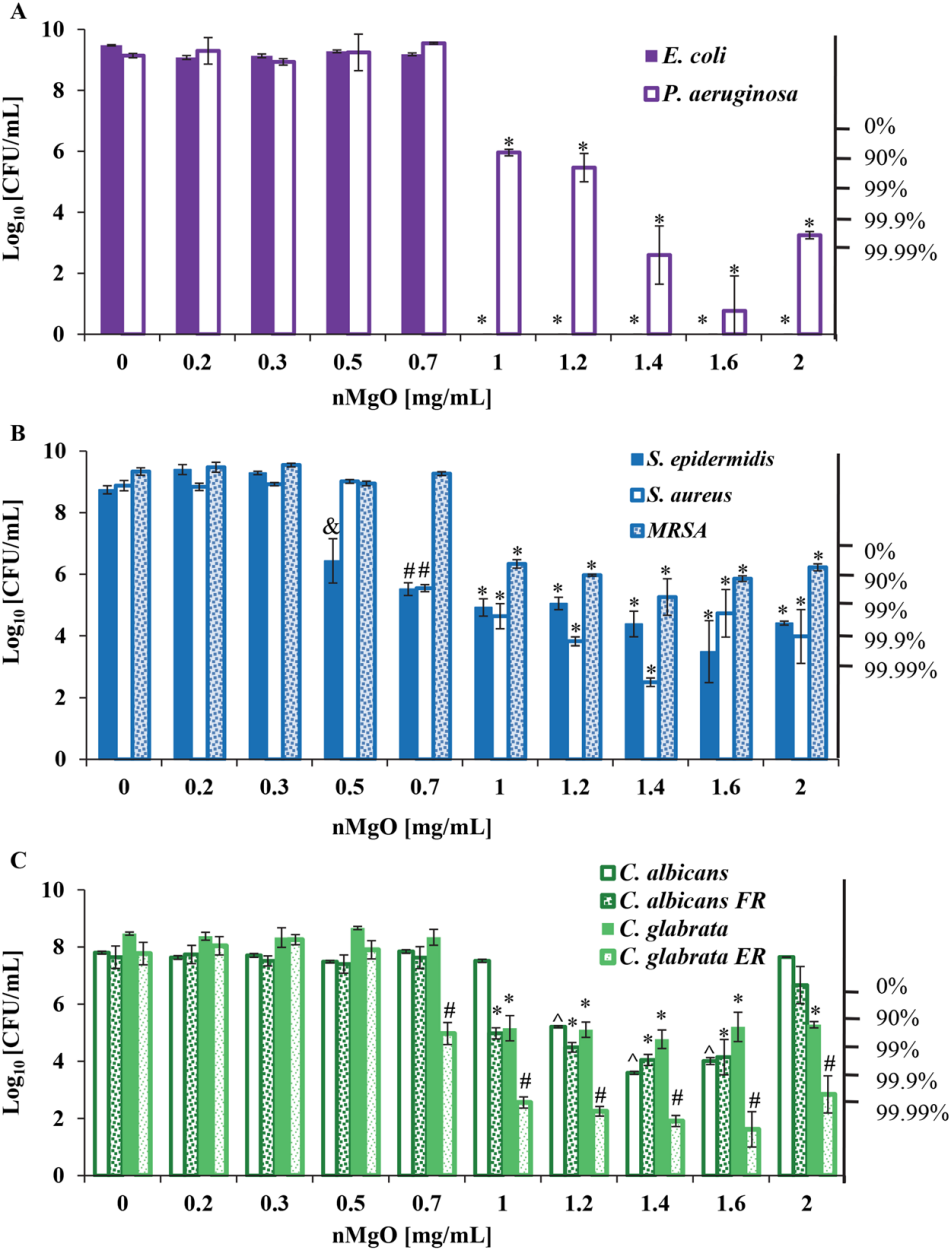
Colony forming units (CFU) quantified for the viable bacteria and yeasts after 24 hours of culture with 0–2.0 mg/mL of nMgO. (**A**) CFUs of gram-negative bacteria, including *E. coli and P. aeruginosa*. (**B**) CFUs of gram-positive bacteria, including *S. epidermidis, S. aureus, and methicillin-resistant Staphylococcus aureus (MRSA)*. (**C**) CFUs of drug-sensitive and drug-resistant fungi, including *C. albicans, C. albicans FR, C. glabrata, and C. glabrata ER*. Data are mean ± standard deviation (N = 9). FR = Fluconazole resistant and ER = Echinocandin resistant. *p ≤ 0.05: significantly lower than the groups at 0–0.7 mg/mL of nMgO for the respective bacterium or yeast. ^*p* ≤ 0.05: significantly lower than the groups at 0–1 mg/mL of nMgO for the respective microorganism. ^#^*p* ≤ 0.05: significantly lower than the groups at 0–0.5 mg/mL of nMgO for the respective microorganism. ^&^*p* ≤ 0.05: significantly lower than the groups at 0–0.3 mg/mL of nMgO for the respective microorganism.

The quantification of colony forming units (CFUs) in Fig. 2 showed that the viability of respective bacteria and yeasts were dependent on the nMgO concentrations. The critical MIC and MLC values of nMgO against each microorganism, are summarized in Table 2. The most potent concentration of nMgO (MPC) in the tested range of 0-2 mg/mL was 1.4 and/or 1.6 mg/mL for all the microorganism tested; and interestingly *E. coli* were completely killed at 1–2 mg/mL of nMgO. The MPC values of nMgO could be valuable for potential clinical translations in the future where a wide spectrum of bacteria and yeasts are involved.

**Table 2:** Summary of MIC and MLC values of nMgO against all the pathogenic bacteria and fungi tested.

Microorganisms		MIC, MLC and MPC of nMgO [mg/mL]					
		MIC	MLC_90_	MLC_99_	MLC_99.9_	MLC_99.99_	MPC
Gram-negative bacteria	*E. coli*	1.0	1.0	1.0	1.0	1.0	1.0–2
	*P. aeruginosa*	1.0	1.2	1.4	1.4	1.6	1.6
Gram-positive bacteria	*S. epidermidis*	0.5	0.7	1.0	1.6	N/A*	1.6
	*S. aureus*	0.7	0.7	1.0	1.2	1.4	1.4
	*MRSA*	1.0	1.4	N/A	N/A	N/A*	1.4
Drug-sensitive and resistant yeasts	*C. albicans*	1.2	1.2	1.4	N/A	N/A*	1.4
	*C. albicans FR*	1.0	1.0	1.4	N/A	N/A*	1.4;1.6
	*C. glabrata*	1.0	1.0	1.4	N/A	N/A*	1.4
	*C. glabrata ER*	0.7	0.7	1.0	1.0	1.2	1.4;1.6

*Indicates the species that never reached 99.99% death rate in the tested range of 0–2 mg/mL of nMgO. MPC is the most potent concentration of nMgO in the tested range of 0–2 mg/mL against each microorganism. MLC is the minimal lethal concentration that refers to MBC for bacteria and MFC for yeasts.

#### MBCs for gram-negative bacteria: E. coli and P. aeruginosa

MgO nanoparticles exhibited bactericidal effects against both *E. coli* and *P. aeruginosa*, as shown in Fig. 2A. However, the MBCs were different for the two gram-negative bacteria. Specifically, 1.0 mg/mL was the MBC_99.99_ of nMgO for *E. coli* and 1.2 mg/mL was the MBC_90_ of nMgO for *P. aeruginosa*. Interestingly, as the concentration of nMgO increased, *E. coli* changed quickly from proliferation at 0–0.7 mg/mL of nMgO to complete death at 1.0–2.0 mg/mL of nMgO; that is, no viable *E. coli* was detected and no CFU was found on agar plates even when there was no dilution. In contrast, as the concentration of nMgO increased, *P. aeruginosa* gradually changed from proliferation at 0–0.7 mg/mL of nMgO to reduction in CFUs at 1.0–2.0 mg/mL of nMgO. Specifically, *P. aeruginosa* was inhibited at 1.0 and 1.2 mg/mL of nMgO and showed over 99.9% of death at 1.4–2.0 mg/mL of nMgO.

#### MBCs for gram-positive bacteria: S. epidermidis, S. aureus and MRSA

MgO nanoparticles showed different inhibitory and bactericidal effects for the three gram-positive bacteria that are in the same genus of *Staphylococcus*, as shown in Fig. 2B. In general, as the concentration of nMgO increased, the CFU of *S. epidermidis* decreased. The growth of *S. epidermidis* was inhibited starting at 0.5 mg/mL of nMgO. The MBC_90_ of nMgO for *S. epidermidis* was 0.7 mg/mL. At 1.6 mg/mL of nMgO, the average death rate of *S. epidermidis* was greater than 99.9% when compared with the initial seeding density of (6–8) × 10^6^ cells/mL. In contrast, higher concentrations of nMgO were required to inhibit the growth of *S. aureus* and MRSA, although the bacterial seeding density was similar. The growth of *S. aureus* was inhibited at 0.7 mg/mL of nMgO, and the MBC_90_ of nMgO for *S. aureus* was also at 0.7 mg/mL. At 1.4 mg/mL of nMgO, the CFU of *S. aureus* reached the lowest and showed a death rate of greater than 99.99%; that is, less than 0.01% of bacteria was viable. Although not statistically significant, at higher concentrations of nMgO, such as 1.6 and 2.0 mg/mL, the average number of viable *S. aureus* was higher than that at 1.4 mg/mL of nMgO; and *S. aureus* still exhibited at least 99% of death rate. Similar trend was observed in *P. aeruginosa*, the average CFU was higher at 2.0 mg/mL of nMgO when compared with that at 1.4 mg/mL of nMgO (Fig. 2A).

MRSA is a methicillin resistant *S. aureus* strain, which was known to be extremely difficult to treat as it can easily become resistant to new antibiotic drugs. Much higher concentrations of nMgO were needed to inhibit the growth of MRSA. Specifically, MRSA growth was inhibited at 1.0 mg/mL of nMgO, which is a much higher MIC than that for *S. epidermidis* and *S. aureus* (Fig. 2B). The MBC_90_ of nMgO for MRSA was 1.4 mg/mL, much higher than that for *S. epidermidis* and *S. aureus*.

#### MFCs for yeasts: C. albicans, C. albicans FR, C. glabrata, and C. glabrata ER

Yeasts are often found in the biofilms on catheters and other medical devices and contribute to the infections*. C. albicans* and *C. glabrata* are two different yeast species in the same genus, and have different characteristics such as the virulence factors and morphology. It was not surprising that these yeasts behaved differently when they were exposed to nMgO, as shown in Fig. 2C. In general, between the two drug-sensitive and drug-resistant *C. albicans* strains, *C. albicans FR* appeared to be more sensitive to nMgO than *C. albicans* at certain concentrations of nMgO, such as 1.0 and 1.2 mg/mL of nMgO. At 1.0 mg/mL of nMgO, *C. albicans FR* exhibited at least 90% of death rate, while the growth of *C. albicans* was not inhibited at the same concentration (Fig. 2C). At the concentrations of 1.2 mg/mL of nMgO, over 90% of *C. albicans* and over 99% of *C. albicans FR* were dead. At 1.4 and 1.6 mg/mL of nMgO, the average death rate of *C. albicans* and *C. albicans FR* were 99% or greater. However, at 2.0 mg/mL of nMgO, both *C. albicans* and *C. albicans FR* showed no reduction in CFUs when compared with their initial seeding density. The greater CFUs of *C. albicans* and *C. albicans FR* at 2.0 mg/mL of nMgO suggested a possible paradoxical effect of nMgO on both strains of *C. albicans* (Fig. 2C).

Similarly, MgO nanoparticles showed higher fungicidal potency against drug-resistant *C. glabrata ER* than drug-sensitive *C. glabrata*, as shown in Fig. 2C. The MFC of nMgO was 0.7 mg/mL for *C. glabrata ER*, and 1.0 mg/mL for *C. glabrata*. At 1.0 mg/mL of nMgO, *C. glabrata ER* showed over 99.9% of death rate, while *C. glabrata* showed over 90% of death rate. At 1.2–1.6 mg/mL of nMgO, *C. glabrata ER* showed over 99.99% of death rate, while *C. glabrata* showed over 90% of death rate. For *C. glabrata*, the percentages of fungal death stayed at 90–99% at 1.2–2 mg/mL of MgO.

### Analyses of post-culture broths

#### The pH of post-culture broths

**Figure 3:**
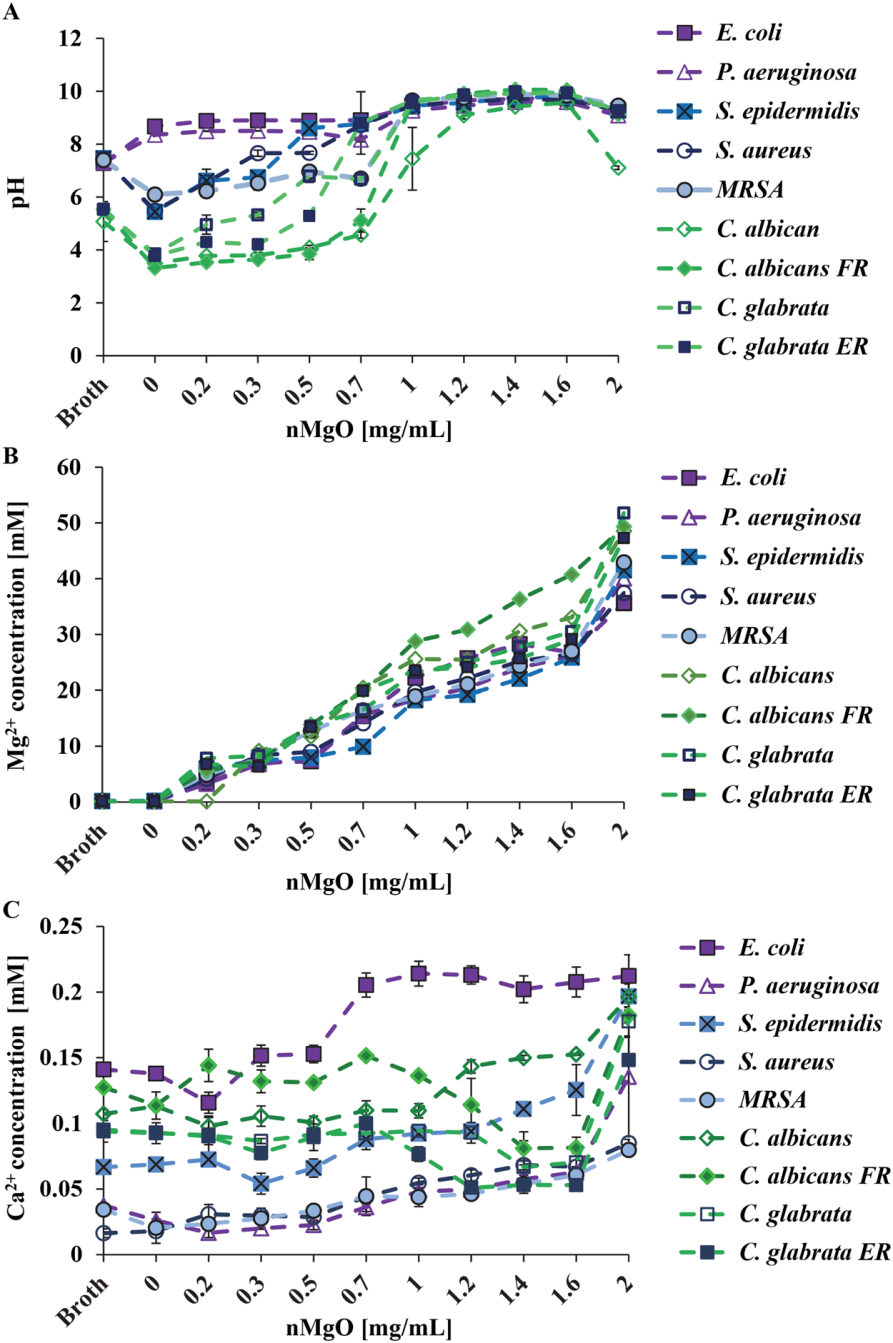
Broth analysis after bacteria and yeasts were cultured with 0–2.0 mg/mL of nMgO for 24 hours. (**A**) The pH of the post-culture broths. (**B**) The concentrations of Mg^2+^ ions in post-culture broths. (**C**) The concentrations of Ca^2+^ ions in post-culture broths. Expanded views of Ca^2+^ ion concentrations are available in Supplementary Materials (Figures S1, S2, S4).

Generally, when the nMgO concentrations increased, the pH of all the broths showed an increasing trend for all of the cultures after 24 hours, as shown in Fig. 3A. For gram-negative bacteria, from 0.2–0.7 mg/mL of nMgO, the pH increased to 8–9. At the concentrations where MICs or MBCs_90_ were observed for *E. coli* and *P. aeruginosa*, the pH increased to above 9. For gram-positive bacteria, the pH started to increase with the addition of nMgO starting at 0.2 mg/mL, when compared with the control group of 0 mg/mL nMgO. However, when gram-positive bacteria multiplied in the absence of nMgO (i.e., the group of 0 mg/mL nMgO), they reduced the broth pH to be more acidic than the broth control (i.e., TSB). When compared with the broth control, the pH increased significantly at 0.5 mg/mL, 0.7 mg/mL and 1.0 mg/mL of nMgO cultured with *S. epidermidis*, *S. aureus*, and MRSA, respectively. Interestingly, the growths of *S. epidermidis*, *S. aureus*, and MRSA were also inhibited at 0.5 mg/mL, 0.7 mg/mL, and 1.0 mg/mL, respectively.

The pH of the post-culture Sab broths for different yeast strains showed similar trends as that of the gram-positive bacteria. Specifically, the pH of the yeast cultures started to increase with the addition of nMgO starting at 0.2 mg/mL, when compared with the control group of 0 mg/mL nMgO. However, when yeasts were cultured in the absence of nMgO (i.e., the group of 0 mg/mL nMgO), they reduced the broth pH from around 5 to less than 4. The Sab broth controls showed pH values around 5, more acidic than TSB or LBB. The pH increased to above 9 at 1.0–2.0 mg/mL of nMgO for almost all the yeasts strains, except for *C. albicans*; the broth of *C. albicans* cultured with 2.0 mg/mL was around 7.1. Interestingly, at 2.0 mg/mL of nMgO, the growth of *C. albicans* also increased (Fig. 3A).

#### Ion concentrations in post-culture broths

Generally, when the nMgO concentrations increased, Mg^2+^ ion concentrations in all the post-culture broths increased after 24 hours of culture, as shown in Fig. 3B. At 0 mg/mL nMgO (i.e., without adding nMgO), the Mg^2+^ ion concentrations in the post-culture broths for all the microorganisms tested were essentially the same as the broth only controls without cells. At 0.2 mg/mL and above, the Mg^2+^ ion concentrations in the post-culture broths were greater than the broth only controls and the 0 mg/mL nMgO groups; moreover, the increasing trends of Mg^2+^ ion concentrations in the cultures with increasing concentrations of nMgO were nearly linear for all the cultures with different microorganisms.

In contrast, the trends for Ca^2+^ ion concentrations in the post-culture broths varied among the different bacterial and yeast strains, as shown in Fig. 3C. In general, Ca^2+^ ion concentrations for the groups at the 2.0 mg/mL of nMgO were higher than the groups at lower nMgO concentrations for the respective microorganisms tested. For *E. coli*, Ca^2+^ ion concentrations showed no significant difference between the broth control and 0 mg/mL nMgO control group after 24 hours of culture; moreover, Ca^2+^ ion concentrations slightly decreased at 0.2 mg/mL nMgO group and then increased at 0.3 to 2.0 mg/mL nMgO groups, when compared with the controls. For *P. aeruginosa*, the Ca^2+^ ion concentrations slightly decreased for the nMgO groups of 0–0.5 mg/mL as compared with the broth control group; however, the Ca^2+^ ion concentrations gradually increased for the groups of 0.7–2.0 mg/mL of nMgO. For gram-positive bacteria of *S. epidermidis, S. aureus*, and MRSA, in general, the Ca^2+^ ion concentrations showed a slight gradual trend of increase with increasing nMgO concentrations with a few exceptions. For *S. epidermidis*, the Ca^2+^ ion concentrations showed a slight drop at 0.3 mg/mL nMgO along the general increasing trend. The Ca^2+^ ion concentrations showed no significant difference between the broth control and 0 mg/mL nMgO control group for *S. epidermidis* and *S. aureus*, but the MRSA control group (i.e. 0 mg/mL nMgO group) reduced the Ca^2+^ ion concentration when compared with its broth control.

For the four yeasts tested, the trends of Ca^2+^ ion concentrations varied for different yeast types (Fig. 3C). Specifically, the Ca^2+^ ion concentrations for *C. albicans* were lower for the groups at 0.2–1.0 mg/mL of nMgO than the broth only control, and then increased at 1.2–2 mg/mL of nMgO. In contrast, the Ca^2+^ ion concentrations in the post-culture broths with *C. albicans FR* fluctuated and showed a shape of spoon. Specifically, the Ca^2+^ concentrations observed at 0.2, 0.7 and 2.0 mg/mL of nMgO were higher than the broth control and the other groups; and the Ca^2+^ ion concentrations for the groups of 1.2–1.6 mg/mL of nMgO were lower. For the two *C. glabrata* strains, Ca^2+^ ion concentrations showed similar trends with a spoon shape; at 1.0–2.0 mg/mL of nMgO, the Ca^2+^ ion concentrations in the cultures with *C. glabrata ER* were less than that for *C. glabrata*.

The correlation between the concentrations of soluble Ca^2+^ ions in the broth and CFUs for gram-negative bacteria, gram-positive bacteria, and yeasts are presented clearly in the Supplementary Materials (Figures S1, S2 and S4).

### Adhesion and morphology of the bacteria and yeasts after exposure to nMgO

**Figure 4:**
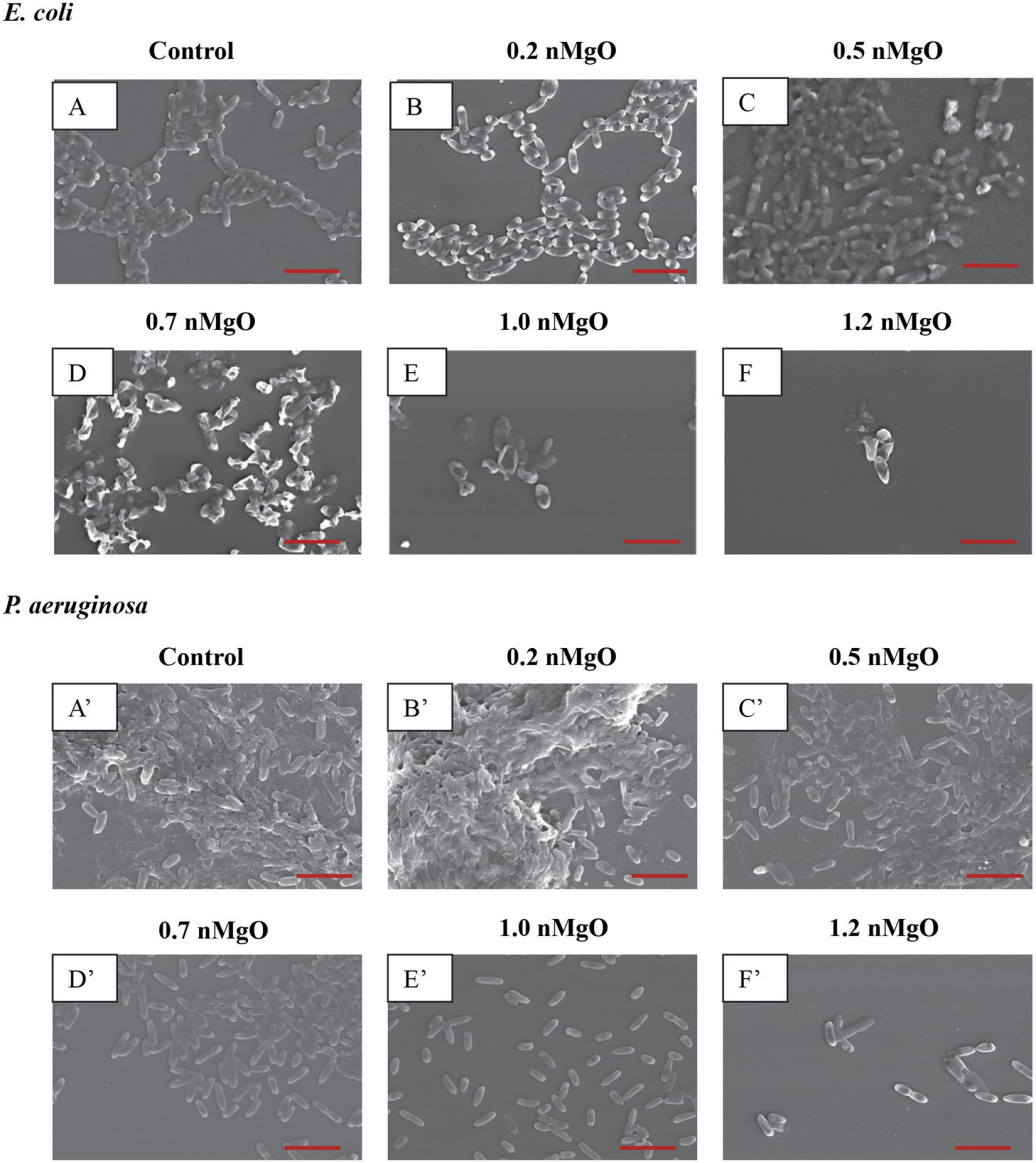
SEM images of gram-negative bacteria, including *E. coli* and *P. aeruginosa*, showing their morphology after they were cultured with 0–2.0 mg/mL of nMgO for 24 hours. Scale bar: 5 µm with an original magnification of 5000x. (**A**) to (**F**) is *E. coli*, and (A)’ to (F)’ is *P. aeruginosa*. The bacteria showed similar morphology at 1.2–2.0 mg/mL of nMgO, and thus only one image at 1.2 mg/mL of nMgO for each bacterial type is shown as a representative for those at the higher nMgO concentrations.

**Figure 5:**
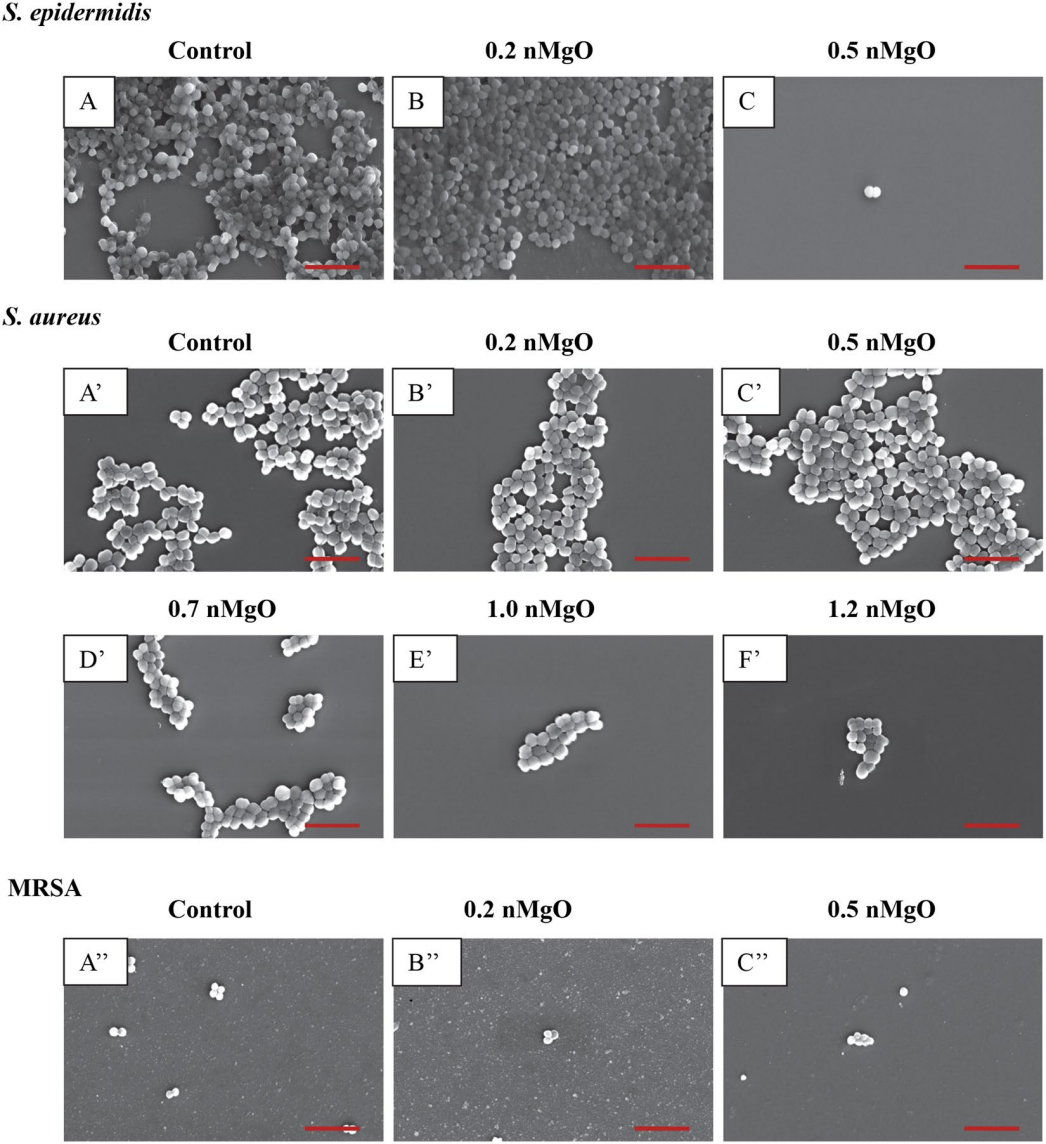
SEM images of gram-positive bacteria, including *S. epidermidis, S. aureus and MRSA*, showing their morphology after they were cultured with 0–2.0 mg/mL of nMgO for 24 hours. Scale bar: 5 µm with an original magnification of 5000x. (**A** to **C**) is *S. epidermidis;* the bacteria at higher concentrations of nMgO were very similar to that at 0.5 mg/mL of nMgO, and only one image is shown as a representative for those at the higher nMgO concentrations. (**A’** to **F’**) is *S. aureus*; the bacteria at 1.2–2.0 mg/mL of nMgO were similar, and thus only one image at 1.2 mg/mL nMgO is shown as a representative for those at the higher nMgO concentrations. (**A”**–**C”**) is MRSA; very few adhered MRSA were found with increasing concentrations of nMgO (none when nMgO concentration >0.5 mg/mL), and thus images are not shown for the higher nMgO concentrations.

**Figure 6:**
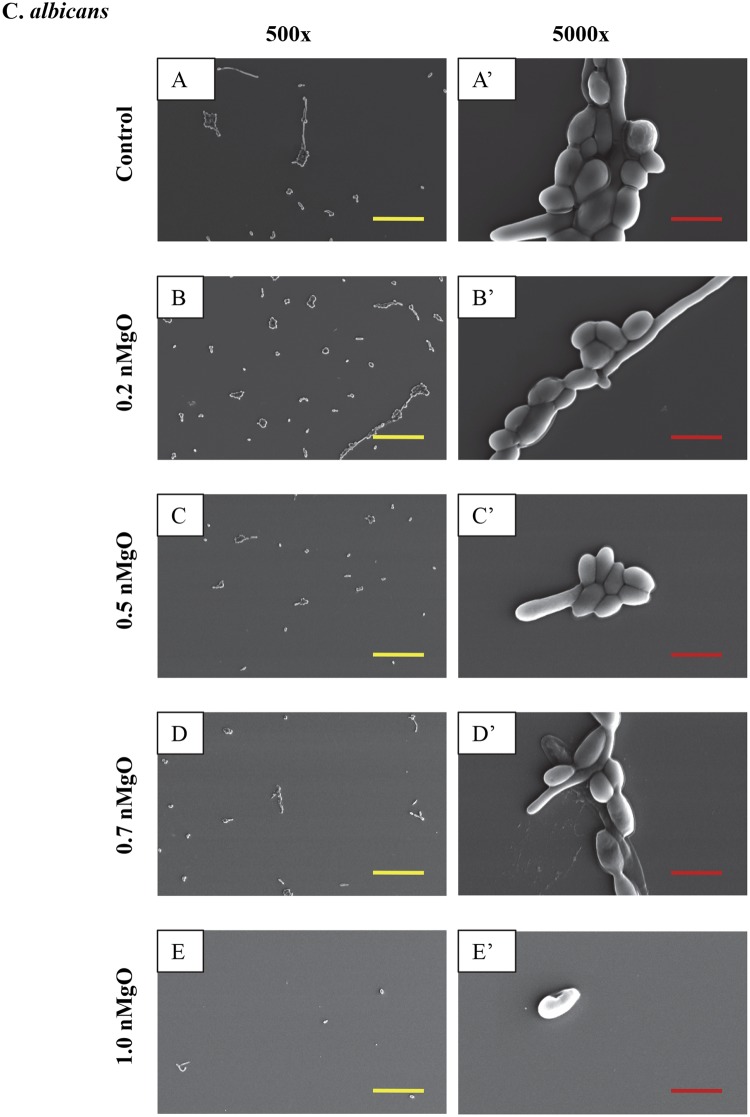
SEM images of *C. albicans*, showing its morphology after 24 hours of culture with 0–2.0 mg/mL of nMgO. (**A** to **E**) Images at an original magnification of 500x with a scale bar of 50 µm. (**A’** to **E’**) Images at an original magnification of 5000x with a scale bar of 5 µm. At 1.0–2.0 mg/mL of nMgO, the cell morphology was similar, and thus only one image at each magnification at 1.0 mg/mL of nMgO is shown as a representative for those at the higher nMgO concentrations.

**Figure 7:**
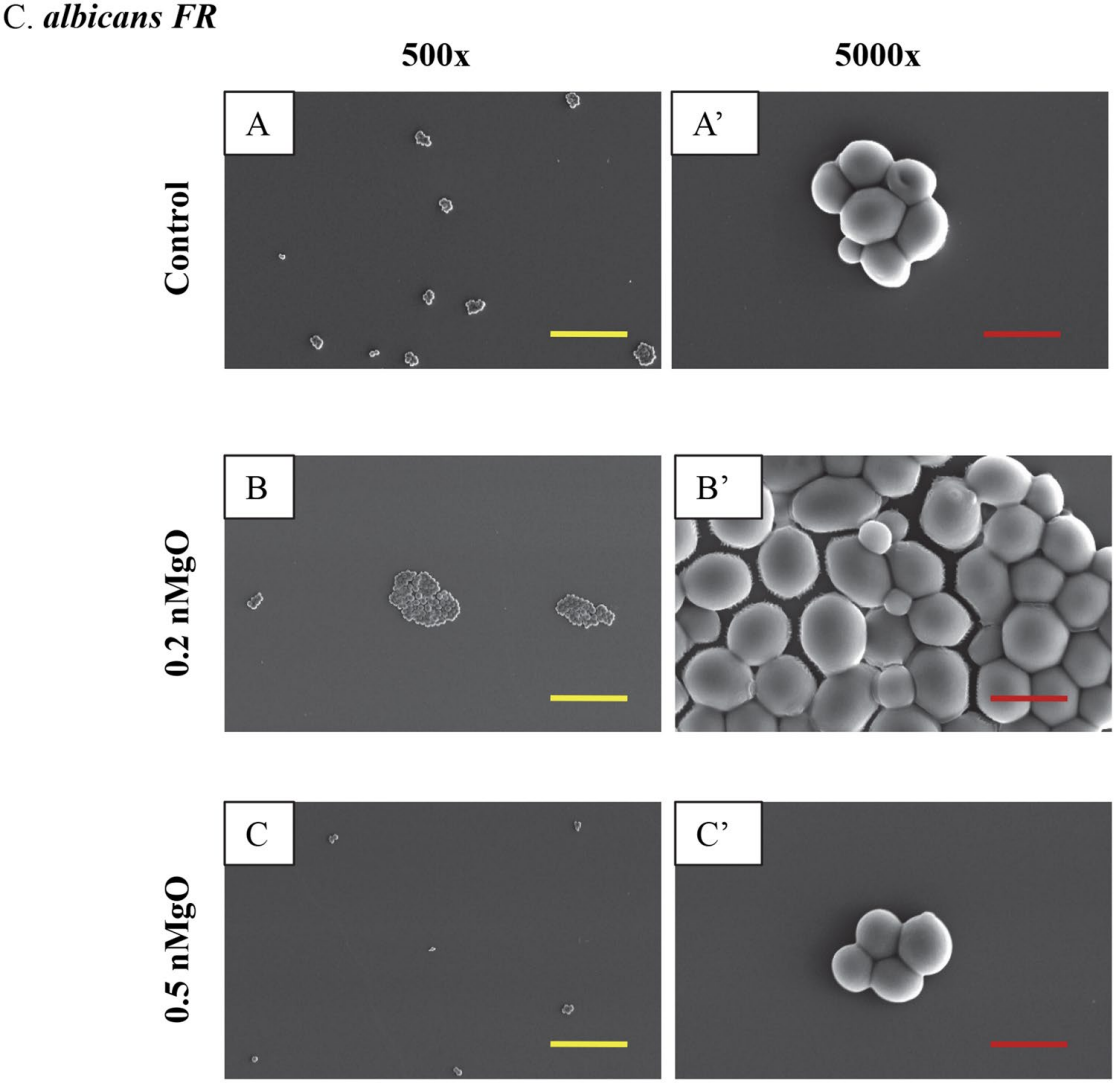
SEM images of *C. albicans FR*, showing its morphology after 24 hours of culture with 0–2.0 mg/mL of nMgO. (**A** to **C**) Images at an original magnification of 500x with a scale bar of 50 µm. (**A’** to **C’**) Images at an original magnification of 5000x with a scale bar of 5 µm. At higher concentrations of nMgO (>0.5 mg/mL), very few adhered *C. albicans* (FR) were found, and thus images are not shown for the higher nMgO concentrations.

**Figure 8:**
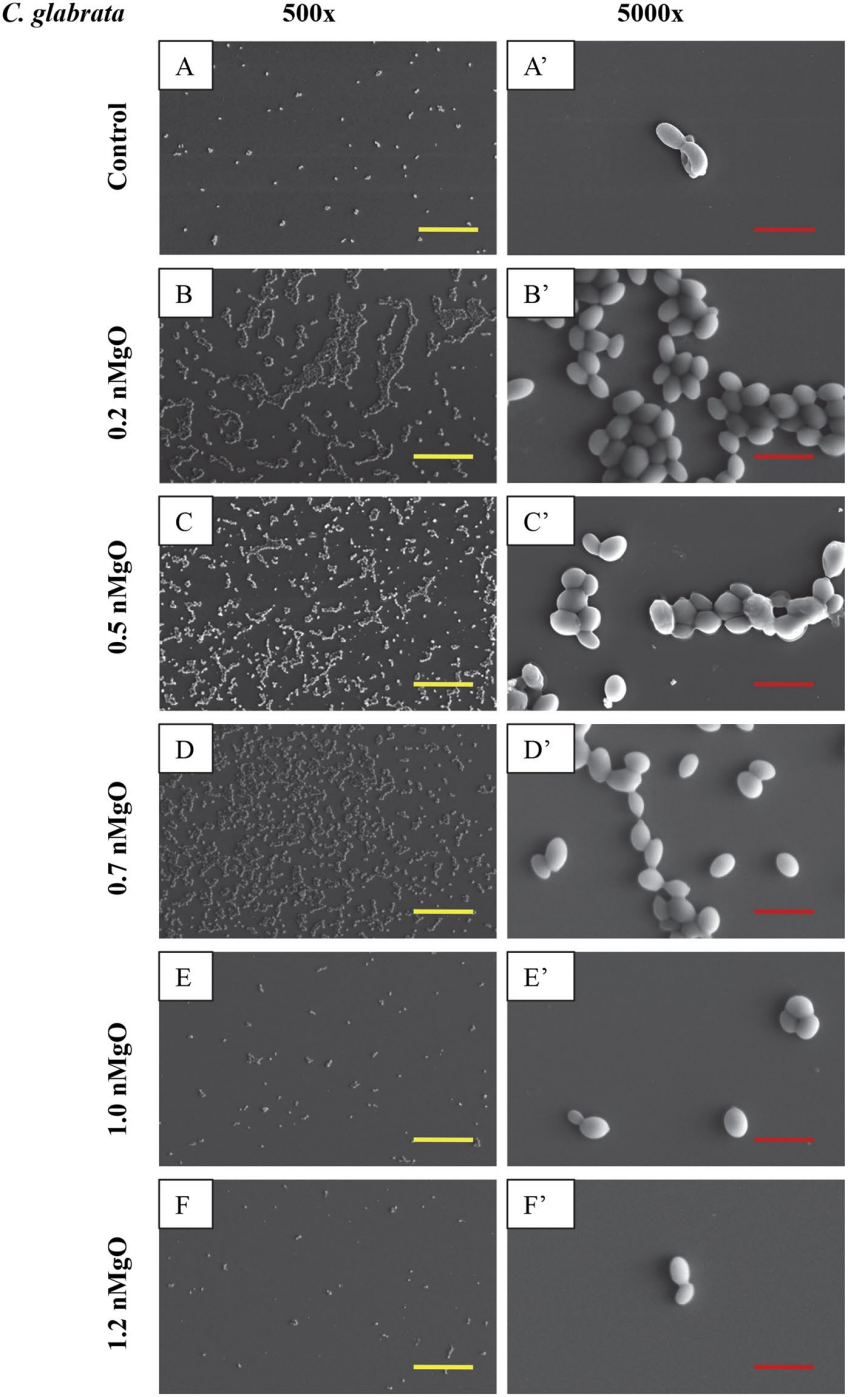
SEM images of *C. glabrata*, showing its morphology after 24 hours of culture with 0–2.0 mg/mL of nMgO. (**A** to **E**): Images at an original magnification of 500x with a scale bar of 50 µm. (**A’** to **E’**) Images at an original magnification of 5000x with a scale bar of 5 µm. At 1.2–2.0 mg/L nMgO, the cell morphology was similar, thus only one image at each magnification at 1.2 mg/mL nMgO is shown as a representative for those at the higher nMgO concentrations.

**Figure 9:**
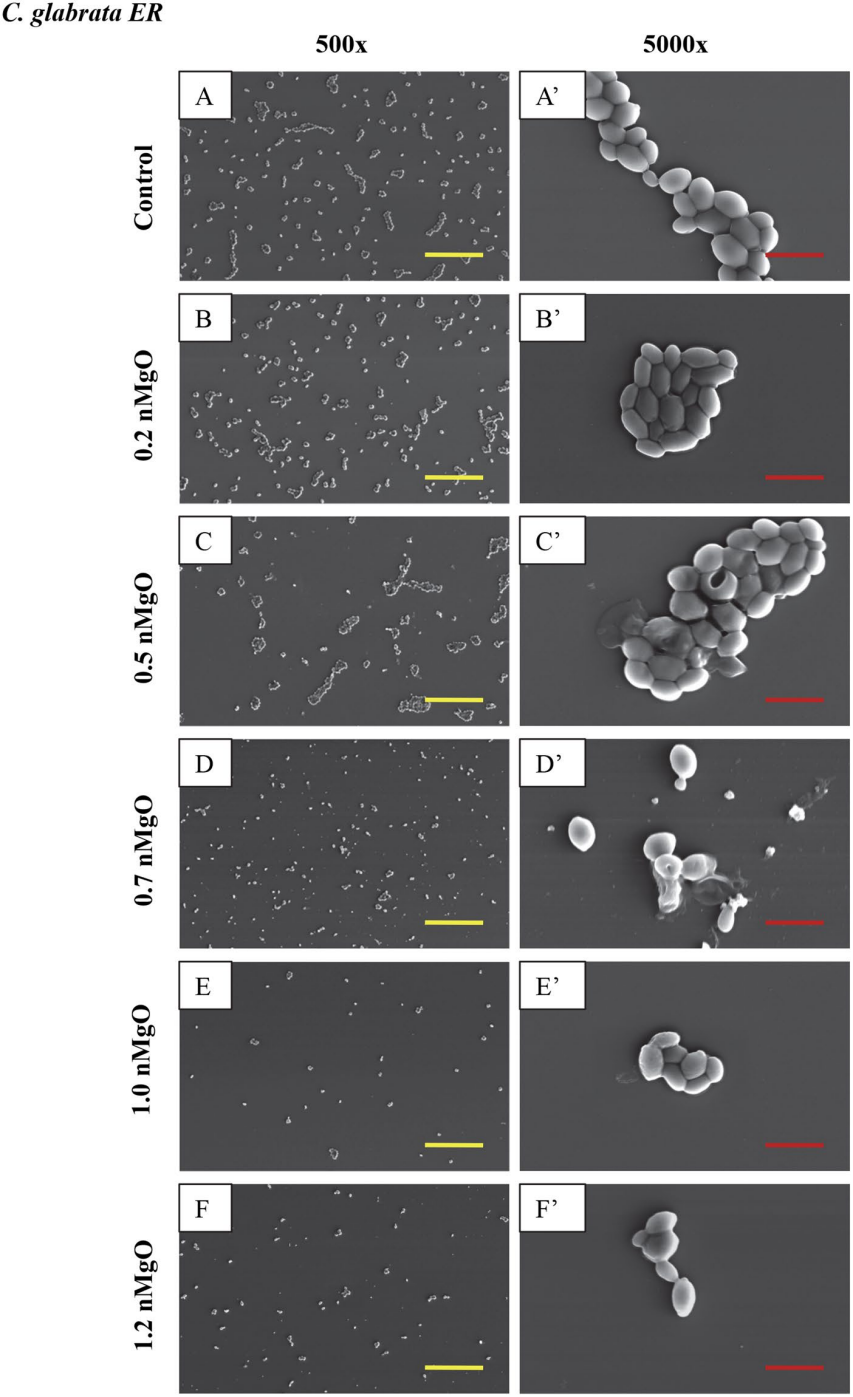
SEM images of *C. glabrata ER*, showing its morphology after 24 hours of culture with 0–2.0 mg/mL nMgO. (**A** to **E**) Images at an original magnification of 500x with a scale bar of 50 µm. (**A’** to **E’**) Images at an original magnification of 5000x with a scale bar of 5 µm. At 1.2–2.0 mg/mL nMgO, the cell morphology was similar, and thus only one image at each magnification at 1.2 mg/mL nMgO is shown as a representative for those at the higher nMgO concentrations.

As shown in the SEM images in Figs 4–9, the nMgO did affect the adhesion and morphology of the bacteria and yeasts of interest, depending on the concentrations of nMgO. In general, when the concentrations of nMgO increased, the adhesion densities of gram-negative bacteria, gram-positive bacteria, and yeasts decreased. Based on the SEM images, nMgO showed different effects on the morphology of each microorganism.

The nMgO particles disrupted the morphology of *E. coli* more obviously than the other bacteria (Figs 4 and 5). At 0.7 mg/mL of nMgO and higher, the shape of *E. coli* was distorted with observable damages in its cell wall and cell membrane (Fig. 4D–F), in contrast to its typical rod shape as seen in the control group (Fig. 4A). The number of *E. coli* attached onto the substrate decreased significantly at the nMgO concentrations of 1.0–2.0 mg/mL; the concentration of 1.0 mg/mL was the MBC of nMgO for *E. coli*. When compared with *E. coli*, no obvious membrane damage was observed for *P. aeruginosa*, although it is also a gram-negative bacterium. Nevertheless, the numbers of attached *P. aeruginosa* decreased when the concentrations of nMgO increased (Fig. 4A’–F’). For the groups at 0 mg/mL (i.e., bacterial control) and 0.2 mg/mL of nMgO, *P. aeruginosa* aggregated with an appearance of a biofilm (Fig. 4A’,B’). At the higher concentrations of nMgO, i.e., 1.0–2.0 mg/mL, not only the number of attached *P. aeruginosa* significantly reduced, but also the attached bacteria were scattered rather than in the form of clusters as seem in a biofilm.

For the gram-positive bacteria, no apparent damage was observed on cell wall or cell membrane when cultured with nMgO, and the cocci (or spherical) shape remained similar to the control group without nMgO (Fig. 5). At 0 mg/mL and 0.2 mg/mL of nMgO, *S. epidermidis* aggregated and showed a morphology similar to a biofilm (Fig. 5A,B). At 0.5 mg/mL of nMgO and above, the number of *S. epidermidis* attached onto the substrate surface decreased significantly (Fig. 5C). As confirmed in Fig. 2, the growth of *S. epidermis* was inhibited at 0.5 mg/mL of nMgO with the CFU lower than the control. For the groups at the nMgO concentrations higher than 0.5 mg/mL, the SEM images for *S. epidermidis* and MRSA were not shown because they were very similar to the groups at 0.5 mg/mL of nMgO, i.e., only a few or no bacteria were found. *S. aureus* formed clusters when attached onto the substrate surface; the clusters were smaller in size with less number of bacteria attached on the surface at the higher nMgO concentrations (Fig. 5A’–F’). A few MRSA attached onto the substrate surface, even in the control group without nMgO (Fig. 5A”). Even fewer attached MRSA were found at 0.2 and 0.5 mg/mL of nMgO (Fig. 5B”,C”), and none was found for the groups with greater than 0.5 mg/mL nMgO. Furthermore, the attached MRSA formed very small clusters with only two to six bacteria.

Yeasts as eukaryotes are typically larger than bacteria, and thus their SEM images were taken at both low and high magnifications (Figs 6–9). The SEM images at the original magnification of 500x were to show a representative overview of the yeast population in a larger field of view, while the images at the original magnification of 5000x were to examine the morphology of yeast cells more closely. Generally, for all the yeast strains tested, more yeast cells attached onto the substrate surface with more and larger clusters at the lower nMgO concentrations. At the higher nMgO concentrations, less yeast cells attached onto the surface, the clusters were smaller, and more yeasts appeared in the form of isolated individual cell. For *C. albicans*, both pseudohyphae and yeast cells were present at the lower nMgO concentrations (i.e., 0–0.7 mg/mL); at 1.0 mg/mL of nMgO and above, very few yeast cells (without pseudohyphae) were found on the substrate and some showed membrane damage (Fig. 6). In contrast to drug-sensitive *C. albicans*, *C. albicans FR* showed mainly oval-shaped morphology and aggregated to form nearly spherical clusters without pseudohyphae (Fig. 7). At 0.5 mg/mL of nMgO and above, very few *C. albicans FR* attached on the substrate. For *C. glabrata*, only oval-shaped yeast cells were observed (Fig. 8), because *C. glabrata* in nature does not form pseudohyphae^36^. Interestingly, when compared with the control group without nMgO (0 mg/mL), the numbers of *C. glabrata* attached onto the substrate surface increased at the nMgO concentrations of 0.2–0.7 mg/mL and decreased when the nMgO concentrations increased to 1.0 mg/mL and above. At 0.5 and 0.7 mg/mL of nMgO, the *C. glabrata* formed smaller clusters or appeared as isolated individual cell when compared with the group at 0.2 mg/mL of nMgO (Fig. 8). When the nMgO concentrations increased to 1.0 mg/mL and above, very few *C. glabrata* cells attached onto the substrate surface. When compared with *C. glabrata*, *C. glabrata ER* showed some damage in membrane as the nMgO concentrations increased (Fig. 9). When the nMgO concentrations increased, the number of attached *C. glabrata ER* decreased, and the clusters were fewer and smaller in size.

### MgO nanoparticles disrupted *S. epidermidis* biofilm

**Figure 10:**
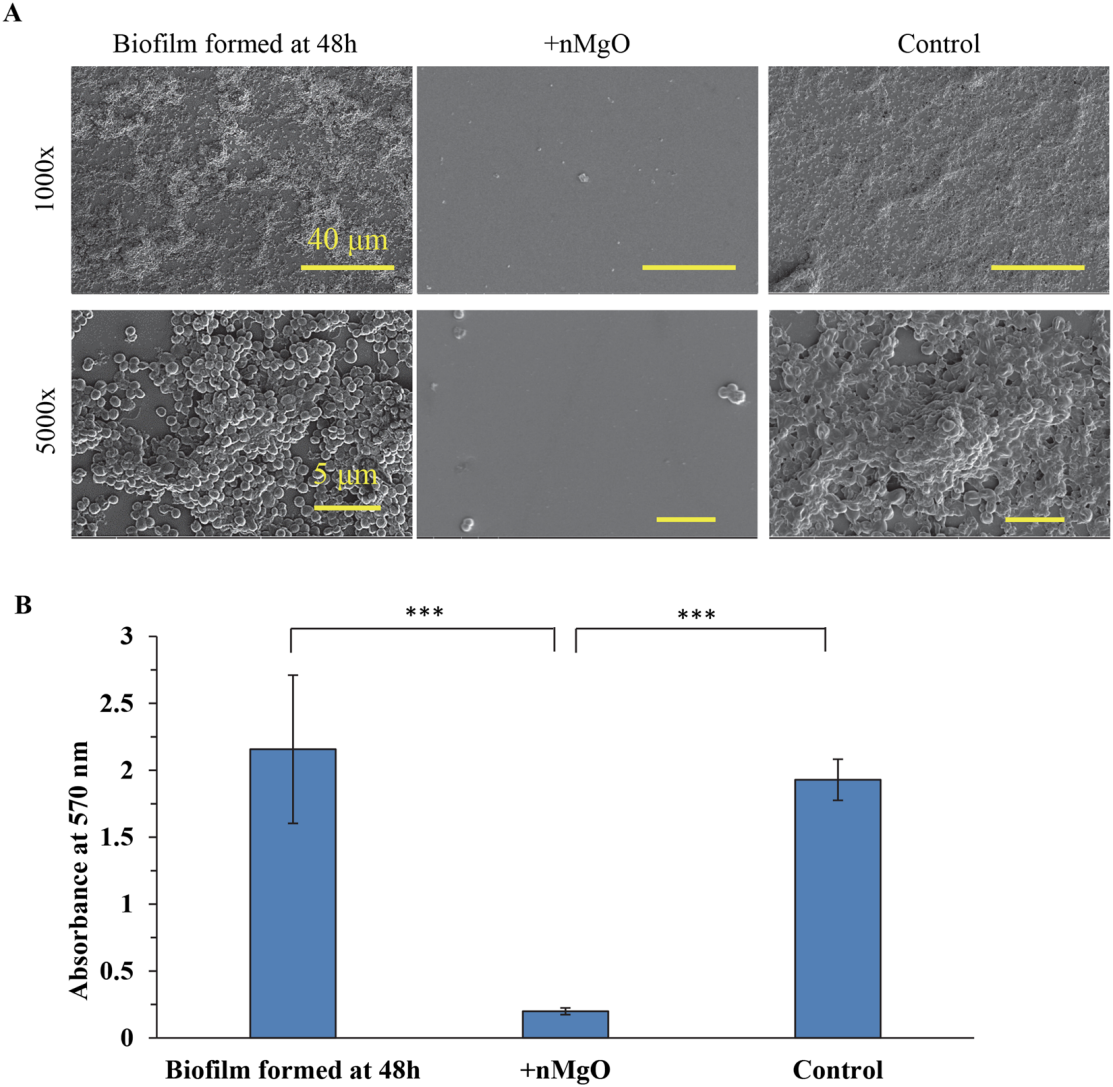
Addition of nMgO disrupted *S. epidermidis* biofilm. (**A**) SEM images of *S. epidermidis* on glass confirmed the formation of biofilm at the 48 hours of culture and the disruption of biofilm when nMgO was added after 48 hours of culture and cultured for additional 24 hours. The original magnification was 1000x with a scale bar of 40 µm and 5000x with a scale bar of 5 µm. (**B**) Crystal violet absorbance at 570 nm. When the biofilm formed after 48 hours of culture, 1.6 mg/mL of nMgO was added into one group and another group served as the control without nMgO being added. Both the group with nMgO (denoted as + nMgO) and the control group without nMgO were cultured for additional 24 hours. Indicates ***p ≤ 0.0005.

Magnesium oxide nanoparticles disrupted *S. epidermidis* biofilm. Before the addition of nMgO, we confirmed that there were indeed biofilms formed on the glass substrate (Fig. 10A). However, at 24 hours after the addition of 1.6 mg/mL of nMgO into the culture, no biofilm was found at both 1000x and 5000x, indicating that nMgO disrupted the biofilm (Fig. 10A). Moreover, very few cells attached onto the substrate; and, the few cells found were in the form of individual cell or small clusters of several cells, as shown in Fig. 10A. In contrast, for the control group where no nMgO was added, the biofilm not only remained, but also grew and secreted more extracellular matrix (Fig. 10A). The absorbance reading of the crystal violet (CV) stain further confirmed the disruption of the *S. epidermidis* biofilm with the addition of nMgO (+nMgO group), when compared with the control group (without adding nMgO) (Fig. 10B). Lower absorbance of CV indicated lower concentration of CV bound to bacteria and thus indicated less bacteria. After the *S. epidermidis* biofilm was cultured with 1.6 mg/mL of nMgO for 24 hours, the nMgO particles were washed away from the culture before the bacteria were stained with CV, which eliminated any possible effects of nMgO on the absorbance readings.

### The effects of pH and Mg^2+^ ions on *S. epidermidis*

**Figure 11:**
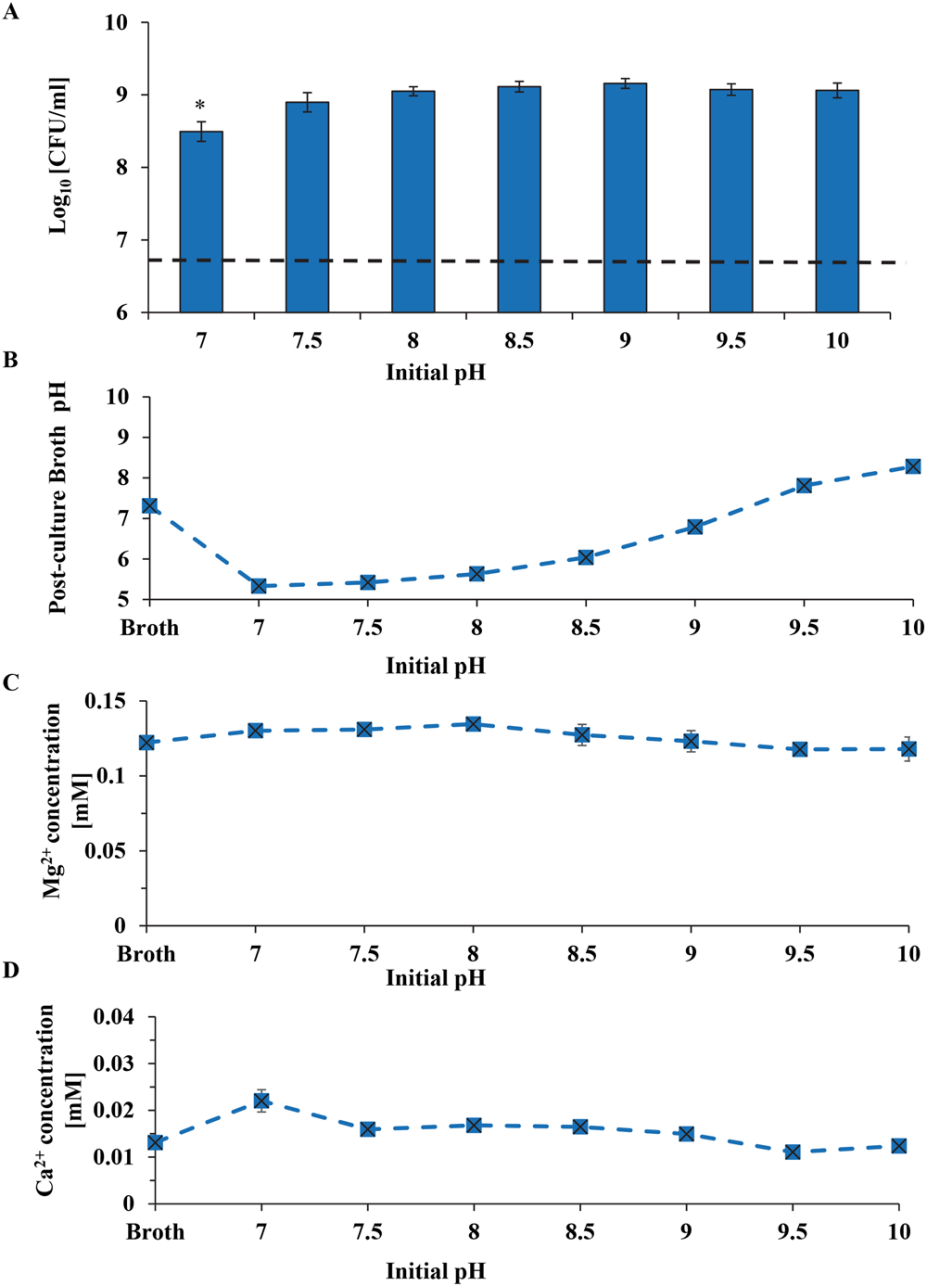
The effects of adjusted initial pH of broths on *S. epidermidis*. (**A**) The CFUs of *S. epidermidis* after 24 hours of culture in the broths with adjusted initial pH values of 7–10. (**B**) Post-culture broth pH. (**C**) Mg^2+^ ion concentrations in the post-culture broths. (**D**) Ca^2+^ ion concentrations in the post-culture broths. Broth indicates the TSB only and serves as a blank reference of broth without bacteria. Dash line in panel A represents the initial *S. epidermidis* seeding density.

**Figure 12:**
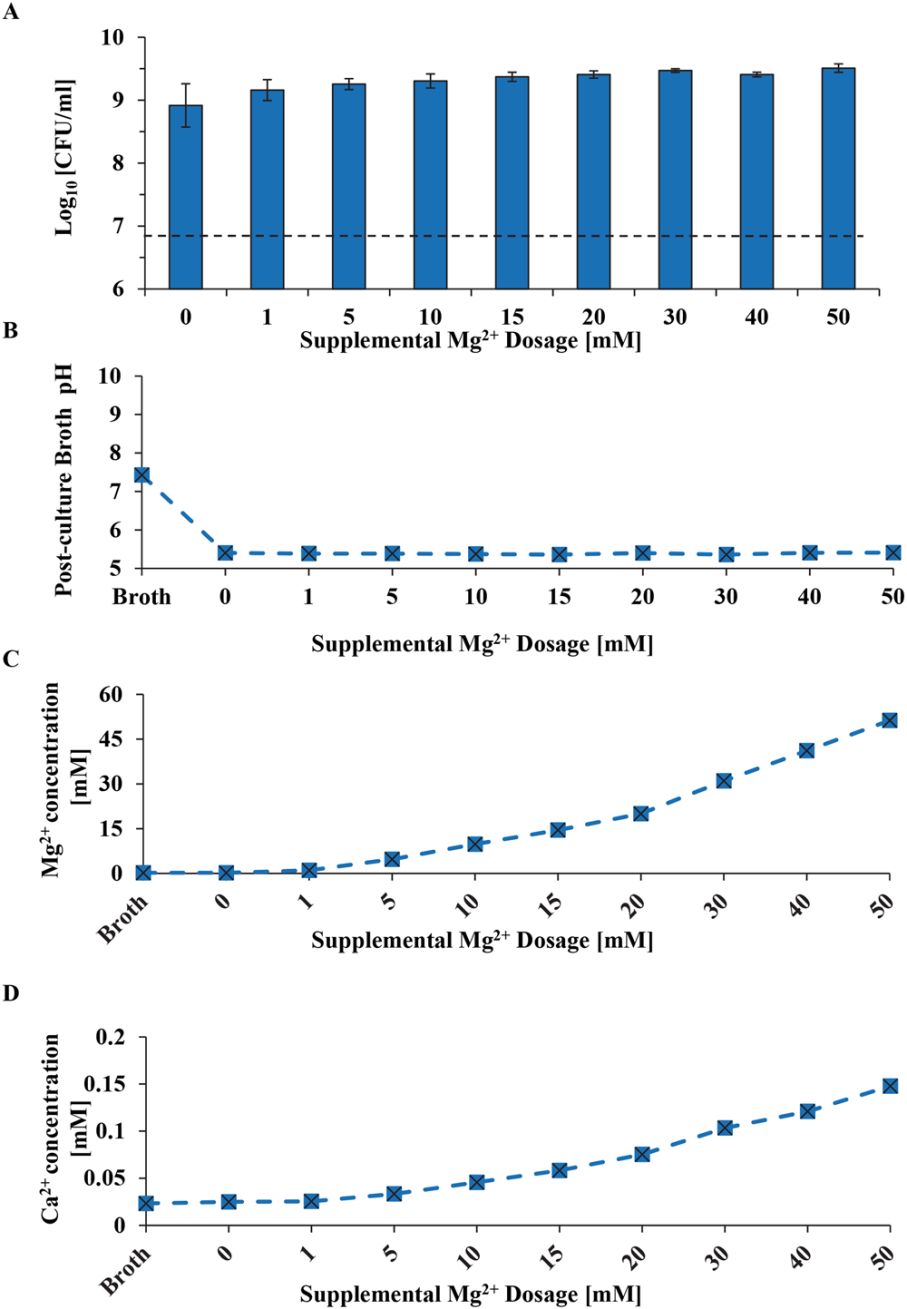
The effects of Mg^2+^ ion doped broths on *S. epidermidis*. (**A**) The CFUs of *S. epidermidis* after 24 hours of culture in the broths with supplemental Mg^2+^ dosages of 0–50 mM. (**B**) Post-culture broth pH. (**C**) Mg^2+^ ion concentrations in the post-culture broths. (**D**) Ca^2+^ ion concentrations in the post-culture broths. Broth indicates the TSB only and serves as a blank reference of broth without bacteria. Dash line in panel A represents the initial *S. epidermidis* seeding density.

Both the higher pH of 7–10 and higher Mg^2+^ ion concentrations up to 50 mM in TSB did not reduce the viability of *S. epidermidis* (Figs 11 and 12). There was bacterial growth in all groups of pH from 7–10; and the CFUs were statistically higher for the groups with the broth pH initially adjusted to 7.5–10 than the control group with the normal broth pH of 7. The results of this pH study confirmed that the alkaline pH was not a contributor to the observed bacterial death when cultured with nMgO, even though the dissociation of nMgO in bacterial cultures caused pH increase. Despite the pH of TSB was initially adjusted to a higher value of 7 to 10 in alkaline region, the post-culture broth pH reduced to 5.3–8.3 respectively when bacteria grew over the 24 hours of culture (Fig. 11B). For example, when the broth pH was initially adjusted to 8.5, the post-culture broth pH reduced to 6. The reduction of post-culture pH confirmed continuous growth of bacteria, because bacteria released acidic metabolites when they grew. In the pH study, the Mg^2+^ concentration remained the same across the different initial pH groups (Fig. 11C) and the Ca^2+^ showed a slight decreasing trend as the initial pH increased (Fig. 11D).

When *S. epidermidis* was cultured in the broth with supplemental dosages of 1–50 mM Mg^2+^ ions, bacteria showed clear growth. The pH of post-culture broth reduced to 5.3–5.4 from the initial pH of 7.3 for all of the groups with supplemental Mg^2+^ dosages, indicating the bacteria continued to grow and release acidic metabolites. Interestingly, in the doped Mg^2+^ ion study, the Ca^2+^ increased (Fig. 12D) as the concentration of Mg^2+^ increased (Fig. 12C). This suggested that Mg^2+^ concentration could affect the Ca^2+^ concentration in the broth. It is important to note that the Mg^2+^ ion concentrations in the post-culture broths were close to the initially doped amount of Mg^2+^ ions.

## Discussions

### Comparability of antimicrobial activities of nMgO against different microorganisms

Antimicrobial properties of MgO have been reported in literature, but these studies involved different experimental techniques, varying size and concentrations of particles, and different concentrations of initial bacterial seeding density^14,15,17,37^. For example, Krishnamoorthy *et al*. reported the minimal inhibition concentration (MIC) of nMgO at the size of 10–30 nm against *Escherichia coli (E. coli), Pseudomonas aeruginosa (P. aeruginosa), and Staphylococcus aureus (S. aureus)* using microtiter plate-based method with a bacteria seeding density of 5 × 10^5^ CFU/mL^17^. Jin *et al*. tested nMgO at the size of 20 nm in the concentrations of 1–8 mg/mL against 10^4^ and 10^8^ CFU/mL of *E. coli* 0157:H57 in a test tube^15^. Jin *et al*. showed that the higher nMgO concentration was more efficient in eliminating food-borne pathogens. Monzavi *et al*. reported fungicidal activity of 10 mg/L nMgO with a size range of 70–150 nm against oral *Candida albicans* (*C. albicans*) using a tube broth dilution assay for endodontic applications^37^. Sawai *et al*. performed a halo test on agar plates to determine the antimicrobial properties of MgO at the size of 3.6 µm and concentration of 100 mg/mL against *E. coli* and *S. aureus* with a seeding density of ~10^9^ CFU/mL^14^. The techniques used in these studies were all different, including microtiter assay, macro dilution assay in test tubes, and agar diffusion assay. The agar diffusion assay involves direct interaction of the nanoparticles with the bacteria on the agar surface, which differs from the interactions of bacteria and nanoparticles in the broth suspensions such as those in microtiter assay and macro dilution assay in test tubes. In the agar diffusion assay, the bacteria are stationary on the surface of the agar, while in broth suspension, the motile bacteria such as *Pseudomonas* species can move. Even though all different methods used by different researchers showed that nMgO is antimicrobial, the results of Sawai *et al*. using agar diffusion assay could not be directly compared with the results from Krishnamoorthy *et al*. who did microtiter assay or Jin *et al*. who performed test tube experiment. Furthermore, different methods were used for quantifying antimicrobial activities of nMgO in these studies, which made the results against different bacterial strains not directly comparable and thus inapplicable toward clinical translation.

The size of nanoparticles may also affect their antimicrobial properties. For example, the smaller silver nanoparticles exhibited higher efficiencies against different organisms^38^. Thus, it is very likely that the size of nMgO would also affect its antimicrobial activities. The antimicrobial properties of nMgO with the size of 20 nm might not be directly comparable with nMgO with the size of 150 nm or 3.6 µm. Moreover, nanoparticles have strong tendency to agglomerate in the broth suspension because of their high surface energy, which could affect the interactions of these nanoparticles with bacteria or yeasts. Despite of constant shaking in the shaker incubator, it is still likely that some nMgO formed agglomerates in the cultures with bacteria or yeasts.

The initial seeding densities of bacteria varied from 10^4^ to 10^9^ CFU/mL in the previous studies^14,15,17,18^, which could be another factor that had played a role in the antimicrobial results. For example, Wetteland *et al*. reported the MIC and MBC_90_ of nMgO was 1.2 mg/mL against *E. coli*, and MIC of 0.7 mg/mL and MBC_90_ of 1.2 mg/mL against *S. epidermidis*^18^, which were greater than the respective values found in this study, even though the same experimental techniques and the same nMgO of the same size were used by Wetteland *et al*. and the only difference was the initial seeding density. Wetteland *et al*. used the seeding density of 5 × 10^5^ CFU/mL, an order of magnitude lower than the seeding density of (6–8) × 10^6^ CFU/mL used in this study. Bacteria could behave differently at different seeding densities because of quorum sensing^39^, which possibly contributed to the different MIC and MBC values reported for nMgO by Wetteland *et al*.^18^. We chose (6–8) × 10^6^ bacteria/mL or yeasts/mL as the seeding density in this study because this was reported to be the critical density causing urinary tract infection, one of the most frequently occurred infections in medical implants and devices^40,41^. However, the exact mechanisms explaining how the bacterial seeding density affected MIC and MBC of nMgO are still unknown and should be further studied to determine if quorum sensing indeed played a key role.

In this study, microorganisms of the same seeding density were tested with nMgO of the same size and same concentration range using the same experimental method, which made it possible to directly compare the antimicrobial properties of nMgO against different microorganisms. Because of these consistent parameters, the results of this study are valuable for translating nMgO toward medical device applications that typically involve a wide range of pathogenic microbes.

### Comparing the antimicrobial activities of nMgO against gram-negative bacteria, gram-positive bacteria, and yeasts

The MBC of nMgO varied for different gram-positive and gram-negative bacteria tested. Gram-positive and gram-negative bacteria mainly differ in their cell wall structure. Gram-negative bacteria have a thin peptidoglycan layer with an outer membrane containing lipopolysaccharides (LPS), while gram-positive bacteria have a thick layer of peptidoglycan without outer membrane and contain teichoic acids. Due to these major differences, each type of bacteria may display diverse sensitivities to antimicrobial agents. When the nMgO concentrations were greater than 1.6 mg/mL, no viable gram-negative bacteria *E. coli* and less than 0.1% viable *P. aeruginosa* were detected, but not in the cases for gram-positive bacteria *S. epidermidis*, *S. aureus* or *MRSA*. Interestingly, the MIC of nMgO was lower for *S. epidermidis* and *S. aureus* than for *E. coli* and *P. aeruginosa*, suggesting that nMgO with increasing concentrations might be inhibitory against the growth of gram-positive bacteria and lethal against gram-negative bacteria.

Unlike bacteria, yeasts are eukaryotic cells. Yeasts have both a fungal cell wall and a cell membrane. Most antifungal drugs target the molecules in the cell wall or the cell membrane^42^. There are three main classes of antifungals: echinocandin, azole and polyenes. Among the antifungals in the class of azoles, fluconazole was widely used for treating infections caused by *C. albicans*^43^. Fluconazole serves as an antifungal drug by interfering the synthesis of ergosterol, a key component in the cell membrane^44^. Resistance to this antifungal drug has emerged due to its excessive usage^43^. We included *C. albicans FR* strain in this study to determine whether or not nMgO would exhibit detrimental effects on the fluconazole resistant *C. albicans* strain. Indeed, not only *C. albicans* but also *C. albicans FR* was sensitive to antifungal activities of nMgO. Interestingly, a possible paradoxical effect on *C. albicans* and *C. albicans FR* was observed; at 2.0 mg/mL of nMgO, the numbers of viable *C. albicans* and *C. albicans FR* (CFUs) were similar to that of the control group without nMgO, indicating no inhibition or killing action against both yeast strains at the highest concentrations of nMgO in this study. A paradoxical effect occurs when the antifungal agent showed no inhibition or killing of the yeasts at the concentrations well above the MIC levels^45^. This paradoxical effect has not been reported for nMgO in literature. The reason for the paradoxical effect is still unknown and this phenomenon should be further investigated in the future.

Most yeast infections are caused *by C. albicans*; however, infections caused by *C. glabrata* have been increasing globally over the past decade, such as healthcare associated candidemia infections^46,47^. *C. glabrata* strains have been reported to develop rapid and increasing resistance to the widely-used antifungal drugs such as the classes of echinocandin, with the increasing use of echinocandin for treating candida infections^46,48,49^. Therefore, we examined the effects of nMgO against drug-sensitive and drug-resistant strains of *C. glabrata*, and found that nMgO was fungicidal to both *C. glabrata* and *C. glabrata ER*. *C. albicans* was generally more resistant to nMgO than *C. glabrata*, which could be due to the ability of *C. albicans* in forming pseudohyphae and hyphae during their growing phase^30^. *C. glabrata* is the only one among all the *Candida* species that does not make pseudohyphae above 37 °C^36^. When *C. albicans* form pseudohyphae during their growth, more nMgO would be required to kill them. In contrast, *C. glabrata* cells retained their oval morphology without forming pseudohyphae when exposed to nMgO, and thus may require less nMgO to kill them. In the SEM images in Fig. 6, there were more yeast cells than pseudohyphae with increasing nMgO concentrations, suggesting that nMgO suppressed surviving yeasts from forming the pseudohyphae. Further research is needed to understand if nMgO inhibits the formation of pseudohyphae.

In summary, Table 2 shows the MICs and MLCs of nMgO at 90%, 99%, 99.9%, 99.99% of killing and the most potent concentration (MPC) of nMgO for all microorganisms studied. Table 2 provides important guidelines for utilizing nMgO in a specific application against a specific microorganism of interest. For example, gram-negative bacteria release lipopolysaccharides (LPS), when they are killed. LPS is an endotoxin that could cause sepsis and inflammation especially at high concentrations, by inducing the production of TNF-α, one of the main pro-inflammatory cytokines^50,51^. In many cases, it may not be desirable to kill the whole bacteria population, but rather to kill enough of them (e.g. 90–99% of death) or inhibit their activities to eliminate the infections without harmful side effects associated with excess and rapid release of LPS. The nMgO may be used in the form of a coating on a medical device or as an additive in a composite material for a medical device, where the nMgO could provide antimicrobial properties against infections without significant side effects. Further studies are needed to determine if the high concentrations of nMgO at MIC, MLC, or MPC level can be integrated into medical devices to achieve desired antimicrobial responses without harming host cells and tissues.

Integration of nMgO with medical devices could provide the devices with sustainable antimicrobial properties while mitigating the toxicity concern regarding burst of high concentrations of nMgO. At lower concentration, the nMgO nanoparticles increased BMSC adhesion density and proliferation^18^, which is beneficial for bone healing and regeneration. However, the nMgO concentrations ranged from 0–2 mg/mL in this study with the most potent concentrations (MPC) of 1.4 and/or 1.6 mg/mL, which are considered high and even toxic to some mammalian cells^18^ if nMgO is used as an antibiotic drug. However, for the clinical applications where nMgO is used in antimicrobial coatings on indwelling medical devices or as an antimicrobial additive in polymer or ceramic based biocomposites for medical devices, this study provided valuable knowledge on the concentration effects of nMgO on the viability of 9 major pathogenic bacteria and yeasts. The results of this study, together with the previous results on the concentration effects of nMgO on BMSCs^18^, presented valuable design guidelines for incorporating nMgO into medical devices and implants, thus reducing implant-associated infections. Even though antibiotics and antifungals have been widely used in clinical practice, post-operative infections still occur, especially involving indwelling medical devices, and many of these infections and associated microbes are drug resistant. So far, there are no reports of microorganisms being resistant to nMgO. Moreover, nMgO is attractive as an additive or coating material for next-generation biodegradable medical devices and implants because nMgO nanoparticles are biodegradable in the body and dissociate into Mg^2+^ ions that are beneficial for tissue healing, in addition to providing desirable antimicrobial properties.

### Possible action mechanisms for nMgO against bacteria and yeasts

**Figure 13:**
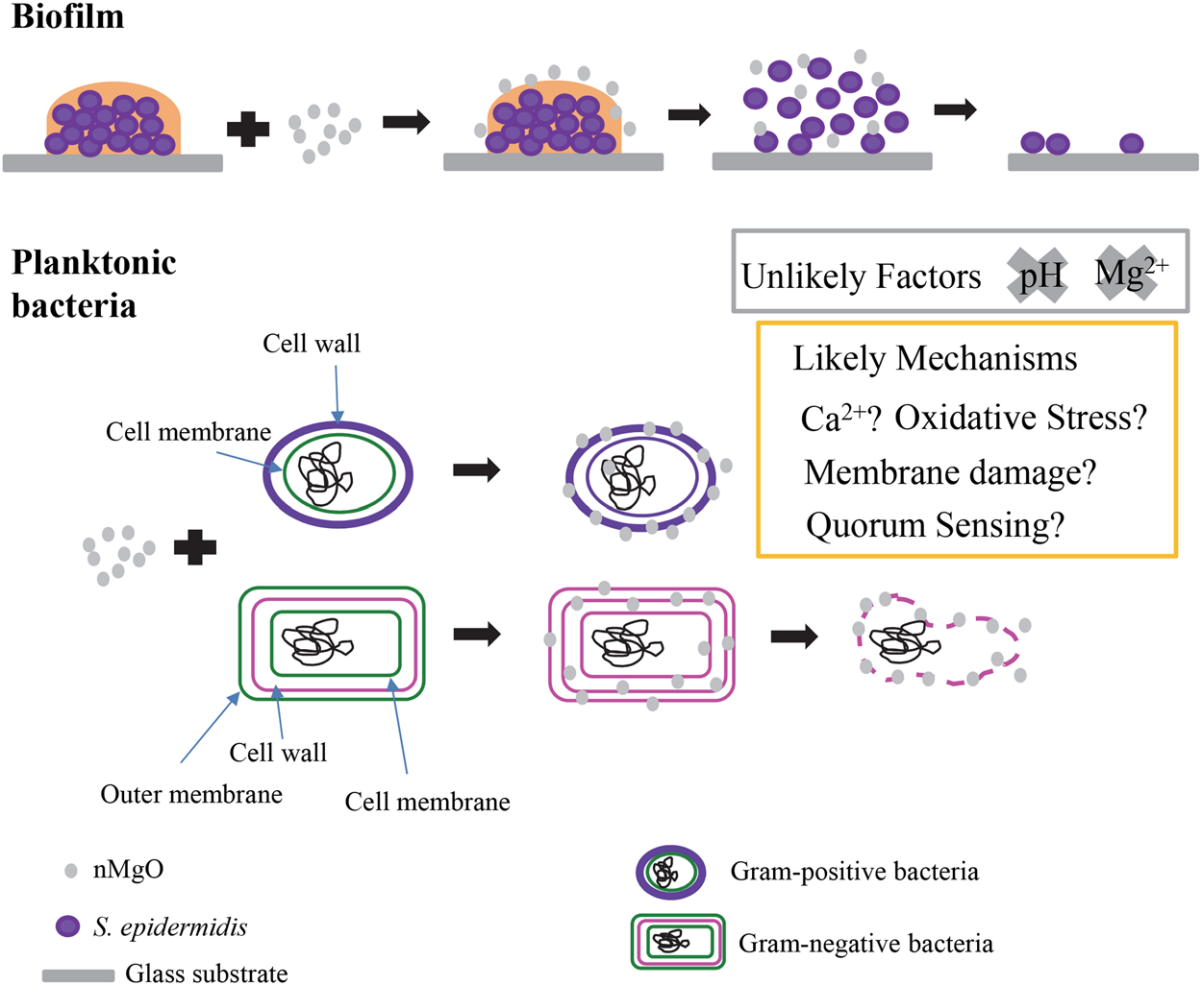
A schematic illustration of the possible mechanisms of nMgO against planktonic bacteria and bacterial biofilm. Oxidative stress, Ca^2+^ ion concentrations, membrane damage, and quorum sensing are the possible mechanisms of nMgO against planktonic bacteria, but alkaline pH of 7 to 10 or increased Mg^2+^ ion concentrations from 1 to 50 mM showed no inhibitory or killing effects on bacteria such as *S. epidermidis*.

The antimicrobial mechanisms of nMgO have been proposed and debated, and currently there is no consensus yet. Figure 13 illustrates the possible mechanisms of nMgO against bacteria. There were some speculations that elevated pH and Mg^2+^ ions could play a role in the action mechanisms of nMgO, considering that nMgO dissociated in bacterial or yeast culture and released OH^−^ ions and Mg^2+^ ions. As the concentrations of nMgO increased from 0–2.0 mg/mL, more nMgO dissociated, resulting in more OH^−^ ions and increased broth pH. However, based on our studies on *S. epidermidis*, the increased pH and Mg^2+^ ions in the broths were not the main contributing factors for antimicrobial activities of nMgO. Specifically, the growth of *S. epidermidis* was not affected when they were cultured in the broths with pH intentionally adjusted to 7–10; and, the deliberate doping of Mg^2+^ ions in the broths up to 50 mM did not cause adverse effects on the viability of *S. epidermidis*. The results reported in literature are in agreement with ours. For example, Sawai *et al*. reported that pH of 10.5 did not affect the growth of *E. coli*^14^. Wetteland *et al*. reported that the pH ranged at 7–10 and supplemental Mg^2+^ ions ranged at 1–50 mM did not affect the growth of *E. coli* and *S. epidermidis*^18^.

Ca^2+^ ion concentrations in the broths showed interesting correlations with the nMgO concentrations, the supplemental Mg^2+^ ion dosages, and the CFUs of bacteria and yeasts (Figures S1–S4 in Supplementary Materials), which suggested that the dissociation of nMgO to Mg^2+^ ions might have played a role on the Ca^2+^ ion concentrations in the cultures. To our knowledge, these correlations have not been previously reported in literature and further research is needed to fully understand the specific roles of Ca^2+^ ions in the bacterial or yeast cultures with nMgO. Additional information is in the Supplementary Materials.

Production of reactive oxygen species (ROS) was proposed as one of the leading theories for the antimicrobial action of nMgO^14,17^. However, Leung *et al*. showed that nMgO inhibited *E. coli* growth without the presence of ROS^52^. Nevertheless, ROS might still be one possible mechanism for the antimicrobial action of nMgO for the following reasons. Bacteria that undergo aerobic respiration will generate superoxide anions and other ROS, and these ROS are toxic to the bacteria. Bacteria also make superoxide dismutase (SOD) to neutralize the ROS^53,54^. The microorganisms studied in this paper are all aerobic, and thus they do produce SOD. The ROS generated by nMgO could be neutralized by the SOD produced by bacteria. However, at higher concentrations of nMgO, more ROS could be produced and not all of the ROS could be timely neutralized by the SOD; and, the excess ROS could still cause damage to the bacteria.

Another antimicrobial mechanism of nMgO could be that nMgO particles bind to the cell membrane to cause damage^15,52^. Membrane damage was observed in *E. coli* when they were cultured with the higher dosages of nMgO (Fig. 4). MgO nanoparticles caused obvious change in the morphology of *E. coli*. The gram-negative bacteria have thinner layer of peptidoglycan, and nMgO could pass the cell wall and bind to the cell membrane, thus causing the shape distortion and cell death as shown in *E. coli*. It is also possible that nMgO particles might accumulate on the thin peptidoglycan layer in the cell wall of gram-negative bacteria and thus damage this thin defense layer of gram-negative bacteria. Whereas in the gram-positive bacteria, considering that the zeta potential of nMgO is positive (Table 1), the positively charged nMgO particles may interact with the negatively charged phosphate groups on the teichoic acid end. If some nMgO did not interact with the phosphate groups, they will be stuck in the thick peptidoglycan layer of the gram-positive bacteria and does not pass through this thick defense layer of gram-positive bacteria to cause further damage. Thus, the membrane damage was observed in the gram-negative bacteria but not in the gram-positive bacteria. No membrane damage was observed in the SEM images of *P. aeruginosa*; however, this does not mean no membrane damage occurred. It is possible that *P. aeruginosa* with membrane damage could not adhere to the glass substrate, thus not shown in the SEM images. When the growth of *P. aeruginosa* was inhibited at the MIC of 1.0 mg/mL nMgO, a small portion of viable *P. aeruginosa* could still adhere onto the glass substrate. Interestingly, nMgO was able to reduce the viability of gram-negative bacteria such as *E. coli* and *P. aeruginosa* to undetectable levels, but it was not the case for the gram-positive bacteria such as *S. epidermidis*. One possible reason is that nMgO caused more severe damage to the cell wall and cell membrane of gram-negative bacteria than to the gram-positive bacteria because of their different structures in the cell walls and membranes. In the case of gram-positive bacteria, nMgO might not be able to pass the cell wall easily due to their thicker peptidoglycan layer. The nMgO particles could be trapped in the peptidoglycan layer of gram-positive bacteria, thus inhibiting their growth. Teichoic acid is another component that extends from the inner side to the outer side of cell wall and exists only in the cell walls of gram-positive bacteria; and their phosphate groups may react with nMgO and trap nMgO on the outer layer of bacterial cell wall^55^. When the nMgO nanoparticles adhered to the outer layer of the cell wall, nMgO could also prevent the adhesion of bacteria onto the glass substrates. For example, when the growth of gram-positive *S. epidermidis* was inhibited at the MIC of 0.5 mg/mL nMgO, there was also a significant reduction in the number of *S. epidermidis* adhered to the glass substrates.

In addition, when nMgO killed the microbes or inhibited the microbial growth, the quorum sensing in the respective microbes might be disrupted considering that the total number of microbes reduced in the culture environment. In other words, nMgO might have disrupted the communications among respective bacteria or yeasts and thus inhibited their activities and functions. Quorum sensing could affect different properties of the bacteria such as virulence factors and the ability of microbes to communicate to each other, as previously described and reported in literature^39,56–61^. If the number of microbes does not reach a certain quorum, their communication is disrupted. The reduction of the number of microbes in culture suspension could prevent the adhesion of respective microbes to a standard substrate, because the communication or coordination of the microbes were disrupted.

There has been little research on antifungal effects of nMgO against yeasts. Both *C. albicans* and *C. glabrata* showed sensitivity to nMgO. The *C. albicans* FR is resistant to fluconazole and fluconazole acts upon the fungal cell membrane by interfering with the synthesis of ergosterol^44^. The yeasts become resistant to azole drugs, either because the drug was pumped out by their efflux pumps, or because the yeasts underwent a point mutation in ERG11 that altered the target protein and thus reduced the binding affinity to the azoles^62^. Considering that nMgO showed the same MIC and MFC against both drug-sensitive *C. albicans* and *C. albicans* FR, the antifungal mechanisms for nMgO might be different from azoles.

While *C. glabrata ER* is resistant to echinocandin, and echinocandin targets the 1,3-β-D-glucan synthase, disrupting the synthesis of 1,3-β-D-glucan, a component in the fungal cell wall. Yeasts can become resistant to echinocandin by having a point mutation in the FKS gene, reducing their sensitivity to echinocandin^49^. The nMgO showed greater efficacy against *C. glabrata ER* strain than against *C. glabrata*, indicating the point mutation in the FKS gene might have made *C. glabrata ER* more sensitive to nMgO. More research is still needed to fully understand how nMgO kill yeasts.

We demonstrated that nMgO could kill planktonic bacteria and disrupt biofilms, suggesting a possibility of another mode of action for nMgO that has not been proposed before in literature. There are three stages in biofilm development on the surface of materials. Initially, the planktonic bacteria adhere to a material or medical device. Next, the adherent bacteria produce the extracellular matrix, proliferate and form the three-dimensional (3D) biofilm structure. Finally, the biofilm releases single or cluster of bacterial cells that can migrate to other areas to start a new site of infection^41^. If the initial step of bacterial adhesion is prevented, no biofilms could form; thus, one way to reduce infection is to reduce bacterial adhesion and/or inhibit their growth on the surface of a medical device. For biofilms that have already formed, the extracellular matrix protects the bacteria inside the biofilm from antibiotics, and therefore, it is much harder to treat infections involving biofilms. However, the biofilm can be treated more easily if it is disrupted. When biofilm is disrupted, dormant cells inside the biofilm become active planktonic cells, which are much easier to be killed by antibiotics than bacteria inside a biofilm. Our results showed that nMgO could kill planktonic *S. epidermidis* and disrupt *S. epidermidis* biofilm. ROS are known to cause protein and DNA damage and thus lead to bacteria death, but extracellular matrix protects the bacteria inside the biofilm from being exposed to ROS^63^. Thus, ROS alone might not be sufficient to kill bacteria inside the biofilm. The disruption of *S. epidermidis* biofilm in this study suggested that nMgO might have other mechanisms of action in addition to ROS or binding to cell membrane of the bacteria. MgO nanoparticles could possibly chelate with proteins and enzymes to disrupt the extracellular matrix, or act as a catalyst to degrade the extracellular matrix of the biofilm. MgO nanoparticles could also diffuse through the water channels in the biofilm matrix and disrupt the internal structure of biofilm. This is the first report on the capability of MgO nanoparticles in targeting and disrupting biofilms; and further studies on the exact mechanisms of nMgO disrupting biofilms are still needed. Different mechanisms of nMgO against planktonic bacteria, yeasts and biofilms are proposed but not fully understood yet. Future studies should focus on mechanistic interactions of nMgO with various pathogenic microorganisms in planktonic forms or in biofilms.

## Conclusions

Magnesium oxide nanoparticles (nMgO) exhibited inhibitory and bactericidal/fungicidal effects on the prevalent pathogenic bacteria and yeasts, as summarized in Table 2. These results are directly comparable across all the microorganisms being studied because we used the consistent experimental methods, the same nMgO in size and shape, and the same initial seeding density for each microorganism of interest. Characterization of cell viability, adhesion and morphology after exposure to nMgO suggested that the nMgO could have different antimicrobial mechanisms against different types of bacteria and yeasts. The increase in broth pH and Mg^2+^ ion concentrations induced by nMgO dissociation was not the main mechanism of killing. The interactions of nMgO with cell wall and/or cell membrane could be the key mechanism for the lethal effects of nMgO against planktonic bacteria. The nMgO was able to eliminate gram-negative bacteria more effectively than gram-positive bacteria, possibly because of the differences in the structures of bacterial cell wall and membrane. This study demonstrated for the first time that nMgO disrupted *S. epidermidis* biofilm, suggesting there might be other mechanisms for nMgO to be antimicrobial. Further research is still needed to determine the exact mechanisms for the bactericidal/fungicidal effects of nMgO against the pathogenic bacteria and yeasts, in order to take full advantage of nMgO for a wide range of applications. This article provides valuable information on nMgO as an antimicrobial biomaterial for engineering infection-free medical devices and implants in the future.

## Electronic supplementary material

Supplementary Materials

Dataset

## Data Availability

All data generated or analyzed during this study are included in this published article and its Supplementary Information files.

## References

[1] Perl TM (2002). Intranasal mupirocin to prevent postoperative Staphylococcus aureus infections. New England Journal of Medicine.

[2] Gao G (2011). The biocompatibility and biofilm resistance of implant coatings based on hydrophilic polymer brushes conjugated with antimicrobial peptides. Biomaterials.

[3] Bryers JD (2008). Medical biofilms. Biotechnology and bioengineering.

[4] Aslam S (2008). Effect of antibacterials on biofilms. American journal of infection control.

[5] Ramage G, Mowat E, Jones B, Williams C, Lopez-Ribot J (2009). Our current understanding of fungal biofilms. Critical reviews in microbiology.

[6] Lynch AS, Robertson GT (2008). Bacterial and fungal biofilm infections. Annu. Rev. Med..

[7] Zimmerli W, Trampuz A, Ochsner PE (2004). Prosthetic-joint infections. New England Journal of Medicine.

[8] Donlan RM (2001). Biofilms and device-associated infections. Emerging infectious diseases.

[9] Peasah Samuel, McKay Niccie, Harman Jeffrey, Al-Amin Mona, Cook Robert (2013). Medicare Non-Payment of Hospital-Acquired Infections: Infection Rates Three Years Post Implementation. Medicare & Medicaid Research Review.

[10] Dizaj SM, Lotfipour F, Barzegar-Jalali M, Zarrintan MH, Adibkia K (2014). Antimicrobial activity of the metals and metal oxide nanoparticles. Materials Science and Engineering: C.

[11] Jones N, Ray B, Ranjit KT, Manna AC (2008). Antibacterial activity of ZnO nanoparticle suspensions on a broad spectrum of microorganisms. FEMS microbiology letters.

[12] Foster HA, Ditta IB, Varghese S, Steele A (2011). Photocatalytic disinfection using titanium dioxide: spectrum and mechanism of antimicrobial activity. Applied microbiology and biotechnology.

[13] Rai M, Yadav A, Gade A (2009). Silver nanoparticles as a new generation of antimicrobials. Biotechnology advances.

[14] Sawai J (2000). Antibacterial characteristics of magnesium oxide powder. World Journal of Microbiology and Biotechnology.

[15] Jin T, He Y (2011). Antibacterial activities of magnesium oxide (MgO) nanoparticles against foodborne pathogens. Journal of Nanoparticle Research.

[16] Stoimenov PK, Klinger RL, Marchin GL, Klabunde KJ (2002). Metal oxide nanoparticles as bactericidal agents. Langmuir.

[17] Krishnamoorthy K, Manivannan G, Kim SJ, Jeyasubramanian K, Premanathan M (2012). Antibacterial activity of MgO nanoparticles based on lipid peroxidation by oxygen vacancy. Journal of Nanoparticle Research.

[18] Wetteland CL, Nguyen N-YT, Liu H (2016). Concentration-dependent behaviors of bone marrow derived mesenchymal stem cells and infectious bacteria toward magnesium oxide nanoparticles. Acta biomaterialia.

[19] Tang ZX, Lv BF (2014). MgO Nanoparticles As Antibacterial Agent: Preparation And Activity. Braz J Chem Eng.

[20] Healey Kelley R., Zhao Yanan, Perez Winder B., Lockhart Shawn R., Sobel Jack D., Farmakiotis Dimitrios, Kontoyiannis Dimitrios P., Sanglard Dominique, Taj-Aldeen Saad J., Alexander Barbara D., Jimenez-Ortigosa Cristina, Shor Erika, Perlin David S. (2016). Prevalent mutator genotype identified in fungal pathogen Candida glabrata promotes multi-drug resistance. Nature Communications.

[21] Taylor P, Schoenknecht F, Sherris J, Linner E (1983). Determination of minimum bactericidal concentrations of oxacillin for Staphylococcus aureus: influence and significance of technical factors. Antimicrobial Agents and Chemotherapy.

[22] Barry, A. L. *et al* Methods for determining bactericidal activity of antimicrobial agents: approved guideline. *NCCLS document M26-A* **19** (1999).

[23] Koul A (2008). Diarylquinolines are bactericidal for dormant mycobacteria as a result of disturbed ATP homeostasis. Journal of Biological Chemistry.

[24] Rao SP, Alonso S, Rand L, Dick T, Pethe K (2008). The protonmotive force is required for maintaining ATP homeostasis and viability of hypoxic, nonreplicating Mycobacterium tuberculosis. Proceedings of the National Academy of Sciences.

[25] Nicolle LE (2012). Urinary catheter-associated infections. Infectious disease clinics of North America.

[26] Campoccia D, Montanaro L, Arciola CR (2006). The significance of infection related to orthopedic devices and issues of antibiotic resistance. Biomaterials.

[27] Makhluf S (2005). Microwave‐Assisted Synthesis of Nanocrystalline MgO and Its Use as a Bacteriocide. Advanced Functional Materials.

[28] Yanagisawa Y, Huzimura R (1981). Interaction of Oxygen Molecules with Surface Centers of UV-Irradiated MgO. Journal of the Physical Society of Japan.

[29] Tian Q, Liu H (2015). Electrophoretic deposition and characterization of nanocomposites and nanoparticles on magnesium substrates. Nanotechnology.

[30] Sudbery P, Gow N, Berman J (2004). The distinct morphogenic states of Candida albicans. Trends in microbiology.

[31] Slonczewski, J. L. & Foster, J. W. *Microbiology: An Evolving Science: Third International Student Edition*. (WW Norton & Company, 2013).

[32] Gu, X., Sun, Y., Tu, K., Dong, Q. & Pan, L. Predicting the growth situation of Pseudomonas aeruginosa on agar plates and meat stuffs using gas sensors. *Scientific reports* **6** (2016). 10.1038/srep38721 PMC5150633 27941841

[33] Weinstein RA, Darouiche RO (2001). Device-associated infections: a macroproblem that starts with microadherence. Clinical Infectious Diseases.

[34] Merritt, J. H., Kadouri, D. E. & O’Toole, G. A. Growing and analyzing static biofilms. *Current protocols in microbiology*, 1B. 1.1-1B. 1.18 (2005). 10.1002/9780471729259.mc01b01s00 PMC4568995 18770545

[35] Brown D, Salt F (1965). The mechanism of electrophoretic deposition. Journal of Chemical Technology and Biotechnology.

[36] Fidel PL, Vazquez JA, Sobel JD (1999). Candida glabrata: review of epidemiology, pathogenesis, and clinical disease with comparison to C. albicans. Clinical microbiology reviews.

[37] Monzavi A, Eshraghi S, Hashemian R, Momen-Heravi F (2015). *In vitro* and *ex vivo* antimicrobial efficacy of nano-MgO in the elimination of endodontic pathogens. Clinical oral investigations.

[38] Agnihotri S, Mukherji S, Mukherji S (2014). Size-controlled silver nanoparticles synthesized over the range 5–100 nm using the same protocol and their antibacterial efficacy. RSC Advances.

[39] Miller MB, Bassler BL (2001). Quorum sensing in bacteria. Annu Rev Microbiol.

[40] Coulthard MG (2010). Redefining urinary tract infections by bacterial colony counts. Pediatrics.

[41] Francolini I, Donelli G (2010). Prevention and control of biofilm-based medical-device-related infections. FEMS Immunology & Medical Microbiology.

[42] Lipke PN, Ovalle R (1998). Cell wall architecture in yeast: new structure and new challenges. Journal of bacteriology.

[43] Albertson GD, Niimi M, Cannon RD, Jenkinson HF (1996). Multiple efflux mechanisms are involved in Candida albicans fluconazole resistance. Antimicrobial Agents and Chemotherapy.

[44] Odds FC, Brown AJ, Gow NA (2003). Antifungal agents: mechanisms of action. Trends in microbiology.

[45] Stevens DA, Espiritu M, Parmar R (2004). Paradoxical effect of caspofungin: reduced activity against Candida albicans at high drug concentrations. Antimicrobial agents and chemotherapy.

[46] Pfaller M (2012). Frequency of decreased susceptibility and resistance to echinocandins among fluconazole-resistant bloodstream isolates of Candida glabrata. Journal of clinical microbiology.

[47] Diekema D, Arbefeville S, Boyken L, Kroeger J, Pfaller M (2012). The changing epidemiology of healthcare-associated candidemia over three decades. Diagnostic microbiology and infectious disease.

[48] Lewis JS, Wiederhold NP, Wickes BL, Patterson TF, Jorgensen JH (2013). Rapid emergence of echinocandin resistance in Candida glabrata resulting in clinical and microbiologic failure. Antimicrobial agents and chemotherapy.

[49] Alexander BD (2013). Increasing echinocandin resistance in Candida glabrata: clinical failure correlates with presence of FKS mutations and elevated minimum inhibitory concentrations. Clinical infectious diseases.

[50] Buttenschoen K, Radermacher P, Bracht H (2010). Endotoxin elimination in sepsis: physiology and therapeutic application. Langenbeck’s archives of surgery.

[51] Kirikae T, Nakano M, Morrison DC (1997). Antibiotic-induced endotoxin release from bacteria and its clinical significance. Microbiology and immunology.

[52] Leung YH (2014). Mechanisms of antibacterial activity of MgO: non‐ROS mediated toxicity of MgO nanoparticles towards Escherichia coli. Small.

[53] Clements MO, Watson SP, Foster SJ (1999). Characterization of the major superoxide dismutase of Staphylococcus aureus and its role in starvation survival, stress resistance, and pathogenicity. Journal of bacteriology.

[54] Karavolos MH, Horsburgh MJ, Ingham E, Foster SJ (2003). Role and regulation of the superoxide dismutases of Staphylococcus aureus. Microbiology.

[55] Neuhaus FC, Baddiley J (2003). A continuum of anionic charge: structures and functions of D-alanyl-teichoic acids in gram-positive bacteria. Microbiology and Molecular Biology Reviews.

[56] Reading NC, Sperandio V (2006). Quorum sensing: the many languages of bacteria. FEMS Microbiol Lett.

[57] Sifri CD (2008). Healthcare epidemiology: quorum sensing: bacteria talk sense. Clin Infect Dis.

[58] Czaran T, Hoekstra RF (2009). Microbial communication, cooperation and cheating: quorum sensing drives the evolution of cooperation in bacteria. PLoS One.

[59] Galloway WR, Hodgkinson JT, Bowden SD, Welch M, Spring DR (2011). Quorum sensing in Gram-negative bacteria: small-molecule modulation of AHL and AI-2 quorum sensing pathways. Chem Rev.

[60] Morohoshi T (2013). Inhibition of quorum sensing in gram-negative bacteria by alkylamine-modified cyclodextrins. J Biosci Bioeng.

[61] Holm A, Vikstrom E (2014). Quorum sensing communication between bacteria and human cells: signals, targets, and functions. Front Plant Sci.

[62] Pfaller MA (2012). Antifungal drug resistance: mechanisms, epidemiology, and consequences for treatment. The American journal of medicine.

[63] Van Acker H, Van Dijck P, Coenye T (2014). Molecular mechanisms of antimicrobial tolerance and resistance in bacterial and fungal biofilms. Trends in microbiology.

