# Supplementary material for: Antimicrobial Activities and Mechanisms of Magnesium Oxide Nanoparticles (nMgO) against Pathogenic Bacteria, Yeasts, and Biofilms
Source: Sci Rep. 2018 Nov 2;8:16260. doi: 10.1038/s41598-018-34567-5 (PMC6214931; doi:10.1038/s41598-018-34567-5)
Supplement: Supplementary file 1 — Supplementary Materials [file 41598_2018_34567_MOESM1_ESM.pdf]

Antimicrobial Activities and Mechanisms of Magnesium Oxide Nanoparticles (nMgO) against Pathogenic Bacteria, Yeasts, and Biofilms

Authors: Nhu-Y Thi Nguyen[1], Nathaniel Grelling[2], Cheyann Lee Wetteland[2], Romeo Rosario[2], Huinan Liu[1,2,3*]

Affiliations: [1]Microbiology Program, [2]Department of Bioengineering, [3]Materials Science and Engineering Program, University of California, Riverside, CA 92521. U.S.A.

*Corresponding Author:

Huinan Liu, PhD
Associate Professor
Department of Bioengineering
Materials Science and Engineering Program
Microbiology Program
University of California at Riverside
900 University Avenue
Riverside, CA 92521
Phone: 951 827 2944
Fax: 951 827 6416
Email: huinan.liu@ucr.edu

Supplementary Materials

Ca^{2+} ion concentrations showed interesting correlations with the nMgO concentrations, the supplemental Mg^{2+} ion dosages, and the CFUs of bacteria and yeasts

Ca^{2+} ions might have played a role in the observed death of bacteria and yeasts. In general, the correlation between the CFU of bacteria and the Ca^{2+} ion concentration showed a shape of "x"; that is, at the concentration of nMgO where the CFUs of bacteria decreased, there was an increase in Ca^{2+} ion concentrations in broth (Figures S1 and S2). For *E. coli*, interestingly, Ca^{2+} ion concentration did not change much at 1.0-2.0 mg/mL of nMgO, possibly because the CFU of *E. coli* reached zero as the amount of nMgO increased to 1.0-2.0 mg/mL (Figure S1). When *S. epidermidis* were cultured in the broth with initial pH intentionally adjust to alkaline region, the Mg^{2+} ion concentration in the broth remained constant and bacterial viability was not affected, but the Ca^{2+} ion concentration slightly decreased as the pH increased (Figure S3). In the doped Mg^{2+} ion study, the viability of *S. epidermidis* was not affected, and the Ca^{2+} concentrations increased as the Mg^{2+} increased. These correlations suggested that both the pH and Mg^{2+} ions might have played a role on the soluble Ca^{2+} ion concentrations in the broths; and the effects of Mg^{2+} was more pronounced than that of pH.

In contrast, the yeasts seem to have a general positive correlation between Ca^{2+} ions and the CFU with a shape similar to a spoon (Figure S4). When the CFUs of yeasts decreased, the Ca^{2+} ion concentration also decreased; and at 2.0 mg/mL of nMgO, the CFUs increased and the Ca^{2+} ion concentration also had a spike. For *C. albicans*, however, the correlation showed some exceptions at 1.2 and 1.4 mg/mL of nMgO. To our knowledge, these correlations have not been previously reported in literature and further research is needed to fully understand these correlations.

Supplementary Figures:

Figure S1: The correlation between the concentrations of soluble Ca^{2+} ions in the broth and CFUs for gram-negative bacteria, including *E. coli* and *P. aeruginosa.*

Figure S2: The correlation between the concentrations of soluble Ca^{2+} ions in the broth and CFUs for gram-positive bacteria, including *S. epidermidis, S. aureus and MRSA*.

Figure S3: The correlation between the concentrations of soluble Ca^{2+} ions in the broth and CFUs for *S. epidermidis* from (A) the pH study and (B) the Mg^{2+} doping study.

Figure S4: The correlation between the concentrations of soluble Ca^{2+} ions in the broth and CFUs for yeast strains, including *C. albicans, C. albicans FR, C. glabrata, and C. glabrata ER*.

Supplementary Figures

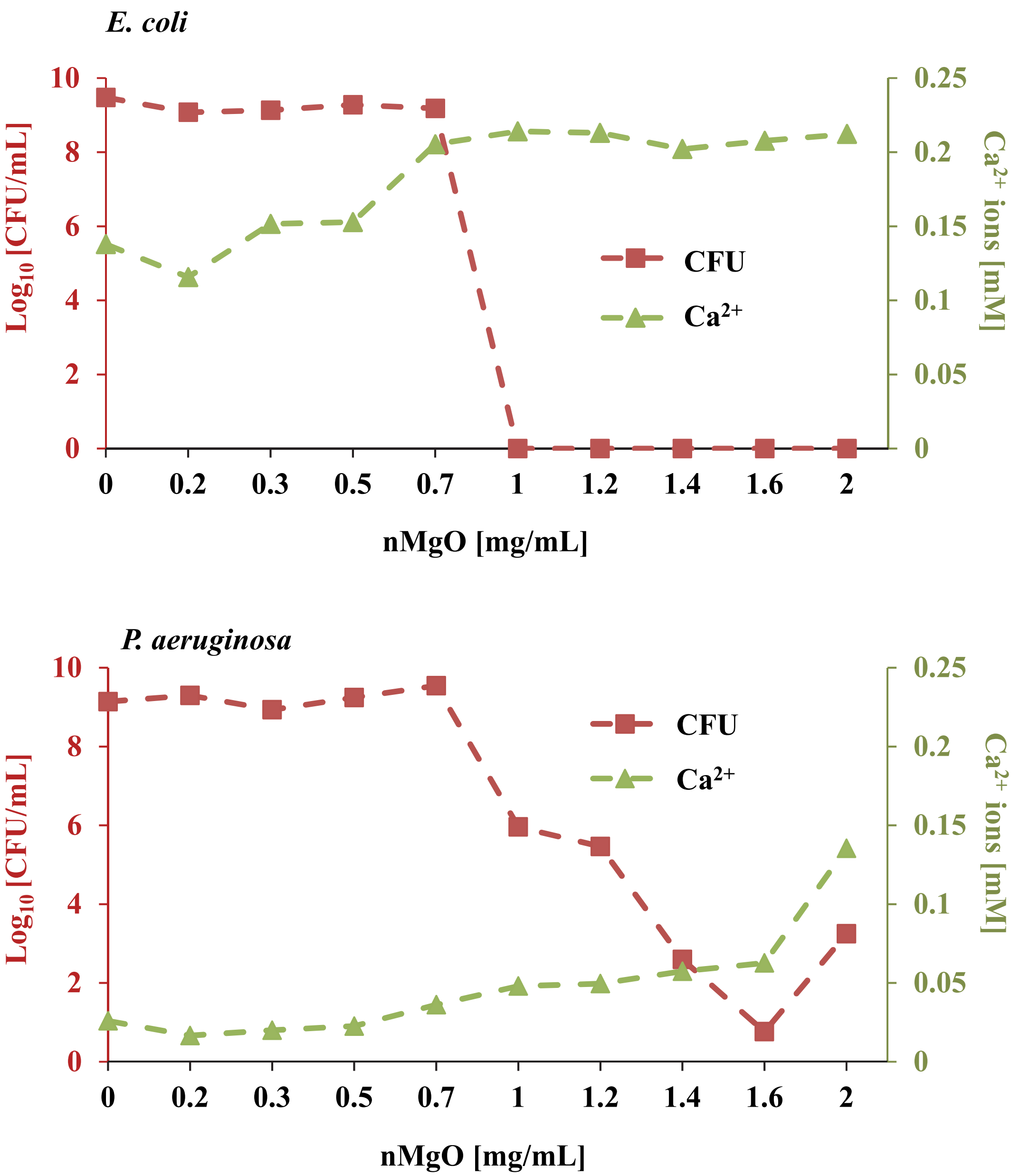

Figure S1: The correlation between the concentrations of soluble Ca^{2+} ions in the broth and CFUs for gram-negative bacteria, including *E. coli* and *P. aeruginosa.*

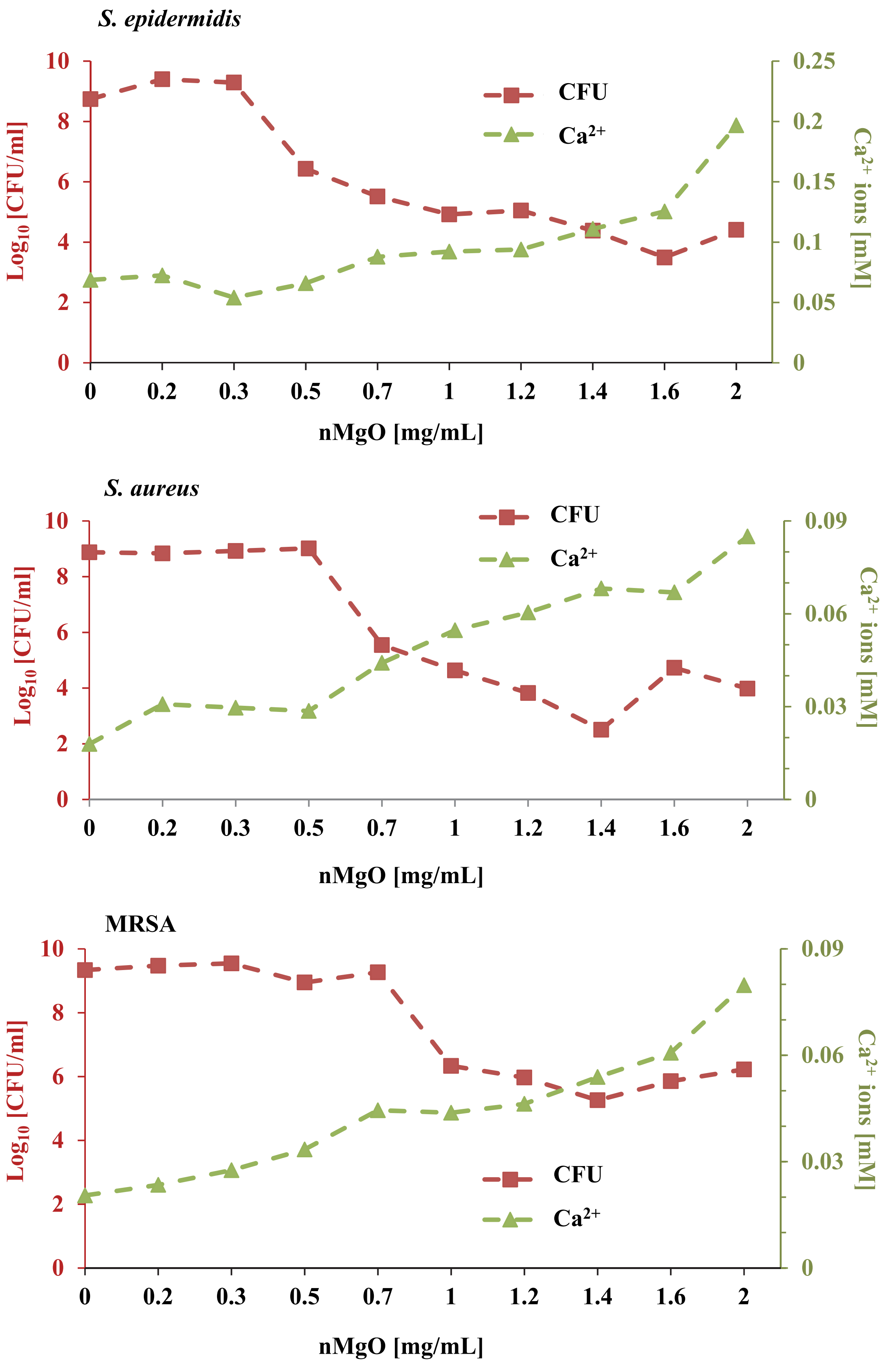

Figure S2: The correlation between the concentrations of soluble Ca^{2+} ions in the broth and CFUs for gram-positive bacteria, including *S. epidermidis, S. aureus and MRSA.*

S. epidermidis

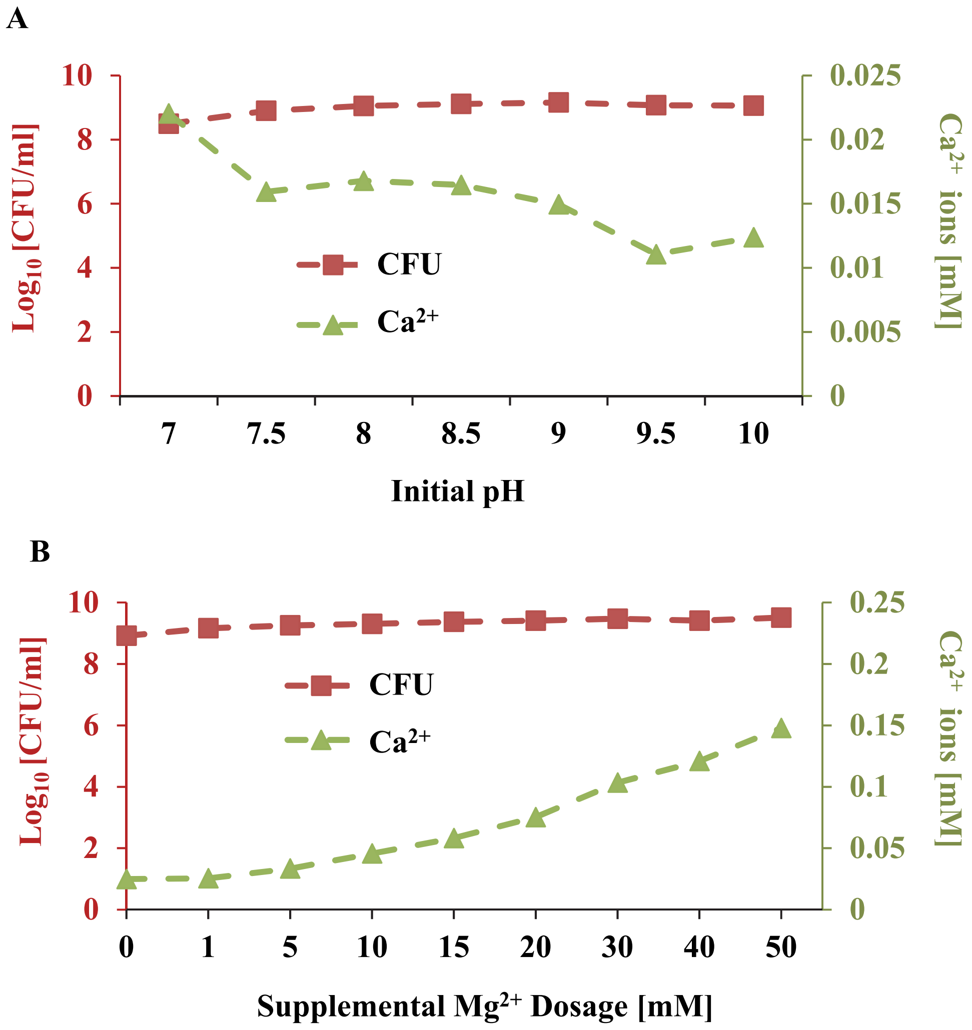

Figure S3: The correlation between the concentrations of soluble Ca^{2+} ions in the broth and CFUs for *S. epidermidis* from (A) the pH study and (B) the Mg^{2+} doping study.

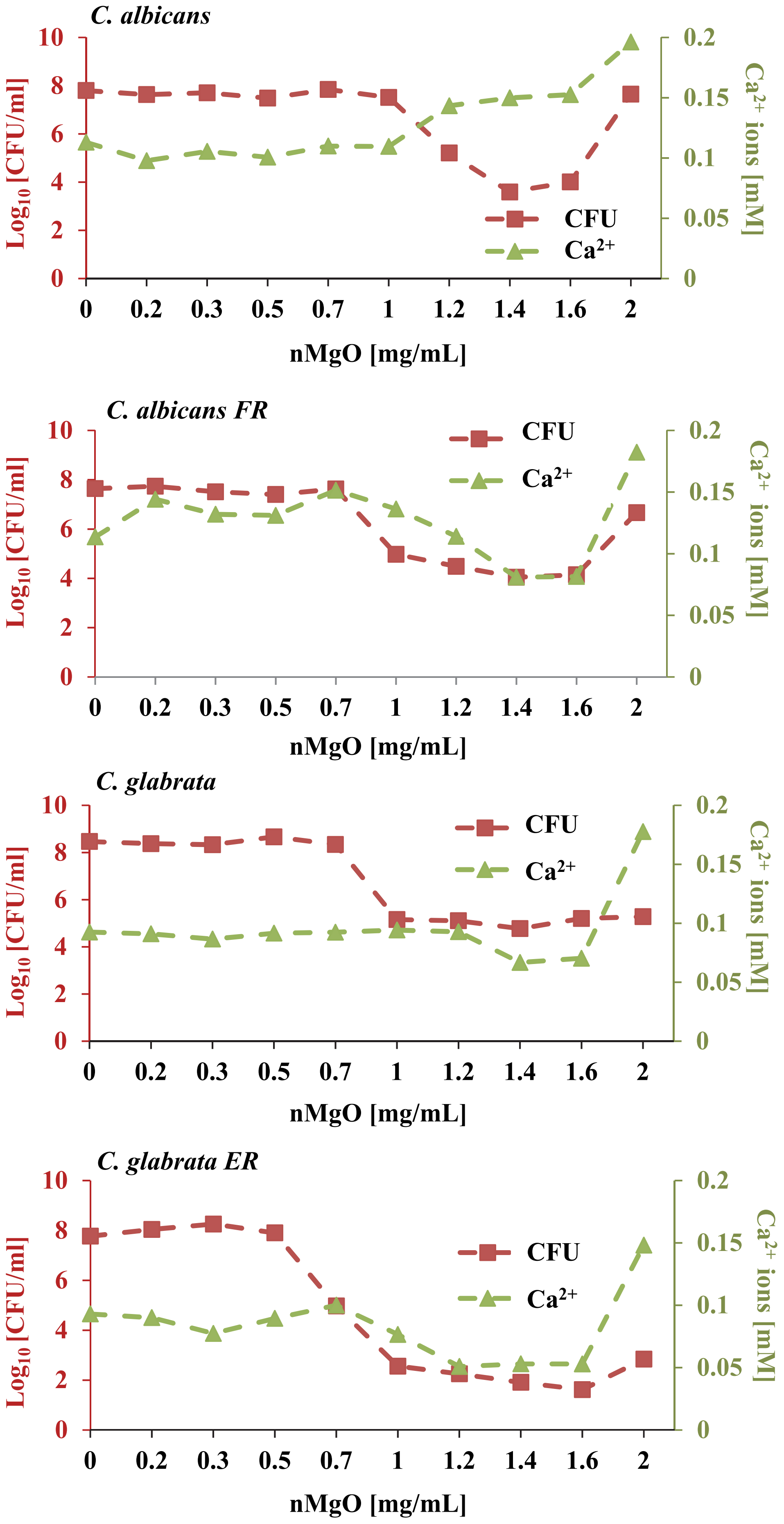

Figure S4: The correlation between the concentrations of soluble Ca^{2+} ions in the broth and CFUs for yeast strains, including *C. albicans, C. albicans FR, C. glabrata, and C. glabrata ER*.
